# Spin density encodes intramolecular singlet exciton fission in pentacene dimers

**DOI:** 10.1038/s41467-018-07736-3

**Published:** 2019-01-03

**Authors:** K. C. Krishnapriya, Palas Roy, Boregowda Puttaraju, Ulrike Salzner, Andrew J. Musser, Manish Jain, Jyotishman Dasgupta, Satish Patil

**Affiliations:** 1 Solid State and Structural Chemistry Unit, Indian Institute of Science, Bangalore, 560012 India; 2 Department of Chemical Sciences, Tata Institute of Fundamental Research, Mumbai, 400005 India; 3 Department of Chemistry, Bilkent University, Ankara, 06800 Turkey; 4 Department of Physics and Astronomy, University of Sheffield, Sheffield, S37RH UK; 5 Department of Physics, Indian Institute of Science, Bangalore, 560012 India; 6 Present Address: Department of Chemistry, Northwestern University, Evanston, IL USA

## Abstract

The formation of two triplet excitons at the cost of one photon via singlet exciton fission in organic semiconductors can potentially enhance the photocurrent in photovoltaic devices. However, the role of spin density distribution in driving this photophysical process has been unclear until now. Here we present the significance of electronic spin density distribution in facilitating efficient intramolecular singlet exciton fission (iSEF) in π-bridged pentacene dimers. We synthetically modulate the spin density distribution in a series of pentacene dimers using phenyl-, thienyl- and selenyl- flanked diketopyrrolopyrrole (DPP) derivatives as π-bridges. Using femtosecond transient absorption spectroscopy, we find that efficient iSEF is only observed for the phenyl-derivative in ~2.4 ps while absent in the other two dimers. Electronic structure calculations reveal that phenyl-DPP bridge localizes α- and β-spin densities on distinct terminal pentacenes. Upon photoexcitation, a spin exchange mechanism enables iSEF from a singlet state which has an innate triplet pair character.

## Abstract (web summary)

Singlet exciton fission – the separation of photoexcited singlet states into two triplet states – holds promise for enhancing photocurrents in photovoltaic technologies. Krishnapriya et al. characterize how electron delocalization over the bridges in a series of pentacene dimers controls this process.

## Introduction

Singlet exciton fission (SEF) is a spin-allowed process that was first discovered in anthracene crystals in 1965^1^. It involves the absorption of one photon by a singlet ground state molecule, followed by separation of the photoexcited singlet excited state into two triplets residing on two different chromophores. SEF as a phenomenon was soon observed also in larger acene structures and discovered later in numerous other conjugated molecules^2–5^. The set of materials that demonstrate SEF is limited by the energy criterion that the singlet state energy (*E*_S_) should be approximately two times or greater than the triplet energy (*E*_T_) i.e. *E*_S_ ≥ 2*E*_T_, though the observation of endothermic fission in tetracene reveals some flexibility in this design principle^5,6^. In addition, the chromophores which satisfy the energy criterion have to be electronically coupled through optimal molecular packing^7,8^. Recently, the dependence of SEF efficiency on molecular packing was circumvented by the demonstration of intramolecular SEF (iSEF) in which the SEF chromophores are coupled via a covalent bridge^9–12^. The orientation of the covalently coupled chromophores plays a significant role in driving iSEF as exemplified by the observation that 1,3-diphenylisobenzofuran shows 200% SEF efficiency in crystals but the quantum yield reduces to 10% when they are covalently coupled^13,14^. Acene dimers have proven more fruitful, with several reports of nearly quantitative intramolecular singlet fission yields in a wide range of structures^11,15–17^. These species are evidence for tunability of rate and yield of iSEF, and even changes in underlying mechanism^16,17^ through the electronic nature of the bonding interaction.

Extensive work has yielded a general consensus that SEF^11,17,18^ proceeds via conversion of photoexcited spin singlet state (S_1_) to dark multi-excitonic coupled triplet pair states (^1^TT), in some cases mediated by charge-transfer states. The intermediate ^1^TT pairs subsequently dissociate to generate pairs of free triplets (T) localized on different chromophores^19–25^. The correlated changes in the electronic states and their corresponding spin configurations in covalently coupled systems have, however, been difficult to comprehend, thereby limiting the design principles. In order to systematically control electron delocalization with spin density distributions, we have synthesized new pentacene dimers with biradicaloid character^24,26,27^ that possess energetically proximate singlet and triplet pair states. The two pentacenes were bridged by push–pull chromophores made of diketopyrrolopyrrole (DPP) linked with three different aryl π-groups: phenyl, thienyl and selenyl, (leading to 2P-PDPP, 2P-TDPP and 2P-SeDPP, respectively). The biradicaloid character of the molecular systems allow singlet to ^1^TT pair mixing through the charge transfer (CT) character of the backbone. Broadband femtosecond transient absorption measurements show that only the phenyl derivative undergoes iSEF, in the picosecond timescale. Theoretical analysis of the spin orbitals and S_1_ states reveal that spin localization and consequently partial ^1^TT character of S_1_, explains the rapid singlet fission of the phenyl derivative.

## Results

### Steady-state electronic spectroscopy

**Fig. 1:**
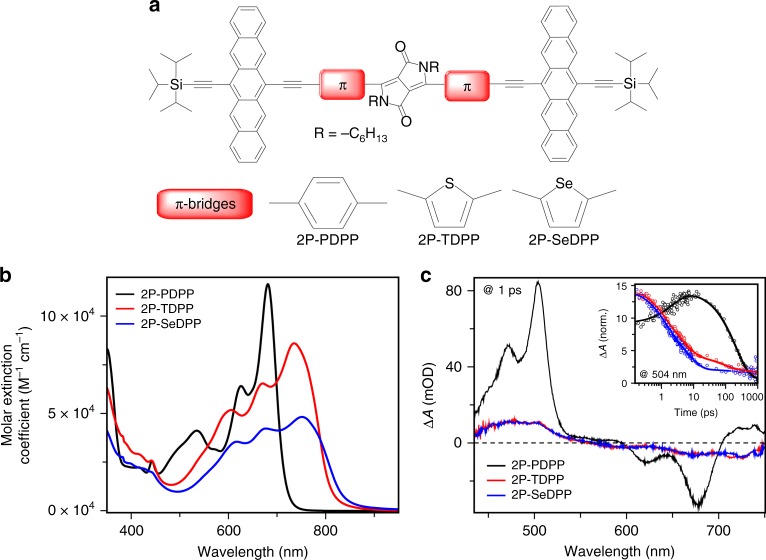
Design of pentacene dimers and their optical properties. **a** Chemical structures of 2P-PDPP, 2P-TDPP and 2P-SeDPP. **b** Steady-state absorption spectra of the three molecules; and **c** their transient absorption spectra (TA) collected at 1 ps pump-probe delay. All three molecules were dissolved in chlorobenzene at 25 μM concentration for the TA measurements, and excited using 0.1 mW pump pulses. For 2P-PDPP the excitation wavelength was 670 nm while it was 650 nm for both 2P-TDPP (red) and 2P-SeDPP (blue). Inset: Transient absorption kinetics taken at the photoinduced absorption peak at 504 nm

The structures of 2P-PDPP, 2P-TDPP, and 2P-SeDPP are given in Fig. 1a. Synthetic procedures and characterization data are available as Supplementary Figures 1, 21–35 and Supplementary methods. UV-visible absorption spectra of the three compounds in chlorobenzene are presented in Fig. 1b. All the three compounds absorb strongly over a broad range of the visible spectrum with contributions identifiable from the pentacene and bridging DPP units (Supplementary Figure 2). The sharp vibronic features of the components seen in 2P-PDPP are significantly broadened in 2P-TDPP and 2P-SeDPP, suggesting conformational heterogeneity. The absorption spectra of 2P-TDPP and 2P-SeDPP are also strongly red-shifted, a consequence of the longer conjugation enabled by the less-bulky 5-membered ring bridges, and exhibit more diffuse absorption. Electronic structure calculations using UTD-B3P86-30%/6-31G* in chlorobenzene reproduced the absorption peak positions with maximum error of 0.20 eV (Supplementary Figure 16). The calculations reveal that the spectral differences of 2P-TDPP and 2P-SeDPP compared to 2P-PDPP are caused by stronger delocalization of the frontier orbitals and smaller energy gaps.

### Excited state dynamics

**Fig. 2:**
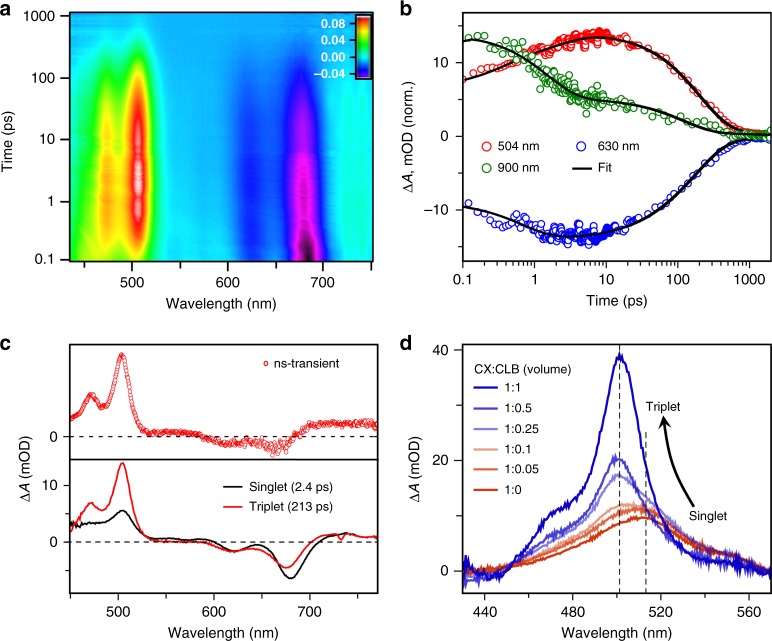
Excited state dynamics of 2P-PDPP and solvent polarity control of SEF. **a** Contour diagram of the obtained broadband transient absorption spectra in between 50 fs to 1ns subsequent to 670 nm excitation. **b** Single-wavelength kinetics at 504, 630, and 900 nm. Symbols are raw data and lines are multi-exponential fits from single-wavelength kinetics analysis. **c** Singlet and triplet species associated spectra obtained from global analysis are compared to the nanosecond transient absorption spectrum experimentally obtained using flash photolysis. **d** The shift and growth of excited-state absorption at 1 ps pump-probe delay with solvent polarity in mixtures of cyclohexane (CX) and chlorobenzene (CLB)

Excited state spectra of 2P-PDPP, 2P-TDPP and 2P-SeDPP in chlorobenzene were investigated using broadband femtosecond transient absorption spectroscopy. In all the samples, we observe positive excited state absorption features (as shown in main Fig. 1c and Supplementary Figure 6) between 450 and 550 nm, while the 550–750 nm spectral region is dominated by ground-state bleach peaks, which match the steady-state absorption. We also observe a broad excited-state absorption in the NIR region from 850 to 1200 nm corresponding to the singlet manifold (Supplementary Figures 5 and 6). In case of 2P-TDPP and 2P-SeDPP, the instantaneously generated S_1_ state exhibits surprisingly fast decay, a major fraction returning to the ground state with no obvious spectral evolution within the first few ps, as evident from the kinetics at 504 nm (Fig. 1c inset) and in the NIR region (Supplementary Figure 7). However, 2P-PDPP exhibits a distinct 504 nm excited-state absorption peak along with a sharp shoulder centered at ~470 nm, thereby being completely distinguishable from the other two molecules (see Fig. 1c). These features are correlated with a unique excited-state absorption peak at 955 nm (Supplementary Figure 5). Moreover, the kinetics of 2P-PDPP at 504 nm shows a rise representing the formation of a new state (inset, Fig. 1c). In order to understand the excited state evolution of 2P-PDPP and to assign the character of the new photoproduct, we carried out detailed investigation of the recorded pump-probe dynamics. The temporal evolution of the excited state spectra is shown as a contour diagram in Fig. 2a. Here, we can distinguish three prominent positive Δ*A* features with peaks at 478, 504, and 730 nm arising from different excited-state absorption transitions. The transient spectral features at 478 and 504 nm rise for ~3 ps while the peak near 730 nm is instantaneously generated (Fig. 2b and Supplementary Figure 5). The 955 nm peak and broad NIR excited-state absorption also form promptly with photoexcitation, with the broad absorption decaying in the sub-ps time scale as shown in Fig. 2b. Following the initial fast process, the remaining excited-state absorption peaks in the visible and NIR regions decay together within ~1 ns.

Global analysis^28^ of the 2P-PDPP TA data set in chlorobenzene reveals two components with lifetimes of 2.4 and 213 ps as shown in Fig. 2c. The first species corresponds to the initially photoexcited S_1_ state, and its decay can be directly resolved in the ESA in the near-infrared kinetics at 955 nm (Fig. 2b). S_1_ converts into the long-lived species characterized by sharp excited-state absorption at 504 nm with a time constant of 2.4 ps. This spectrum is reminiscent of triplet signatures in other pentacene materials and the sharp peak at 955 nm closely matches the triplet absorption of TIPS-pentacene in solution^15,16,29,30^. In order to confirm the assignment to triplets, we performed flash photolysis. As shown in Fig. 2c, the spectrum obtained at 50 ns (red dots) matches well with the ~213 ps component in the transient absorption data. The species in flash photolysis exhibits faster decay in the presence of atmospheric O_2_ (Supplementary Figure 4), confirming that the long-lived species following direct excitation of 2P-PDPP correspond to triplet excitons. Interestingly, the characteristic triplet peak in the NIR is evident from the earliest time delays (Supplementary Figure 5), suggesting some triplet character in the initial photoexcitation; we return to this point below. In the absence of O_2_, the triplet life time of 2P-PDPP solution in chlorobenzene is 8.6 μs. It is evident that some of the triplets formed by iSEF survive the fast (213 ps) recombination regime and persist on much longer timescales, similar to other bridged pentacene dimers^11,15,31^. Moreover, the sensitivity to O_2_ reveals that the triplet energy is between 0.8 and 1 eV, similar to earlier reports of modified triplet energies in orthogonal pentacene dimers^32^.

To characterize the process, we have performed control experiments on pump power, solvent polarity etc. None of the observed behavior was found to change with excitation density, from 0.04 to 0.2 mW pump power (Supplementary Figure 8). Likewise, the kinetics are identical over the concentration range 10–100 μM (Supplementary Figure 9) and DOSY-NMR (Supplementary Figure 12) reveals no appreciable aggregation of 2P-PDPP at the concentrations used for TA, confirming that the process observed is indeed intramolecular. Interestingly, the dynamics are affected by the choice of pump photon energy. When we selectively excite the PDPP bridging unit at 485 nm, we observe a markedly slower rise of the triplet ESA at 504 nm (8.6 ps rather than 2.4 ps, spectra and kinetics are shown in Supplementary Figure 10). This indicates that iSEF is only operative when the DPP-centric singlet state relaxes to the coupled pentacene S_1_-state, thereby sensitizing the process beyond the conventional absorption window of the individual pentacenes. The process of excitation energy transfer through the bridge certainly is an exciting direction for designing new iSEF molecules which feature π-bridges as implicit chromophores exclusively.

### Solvent-dependent singlet fission

In order to probe the mechanistic pathway of iSEF and particularly the role of CT states in mediating the coupling^16^, we have performed solvent-dependent TA of 2P-PDPP. We have used a range of solvents including chloroform, chlorobenzene, toluene and cyclohexane as well as two solvent mixtures. Figure 2d shows a comparison of transient absorption spectra of 2P-PDPP at 1 ps pump-probe delay in a range of mixtures of chlorobenzene and cyclohexane. In pure cyclohexane, only the S_1_ signature can be observed on these timescales; indeed, no iSEF is observed in cyclohexane even at much longer delays (Supplementary Figure 13). It is evident that the sharp triplet state absorption near 504 nm emerges gradually as the polarity of the medium increases.

### Multiexcitonic bright state of 2P-PDPP

To establish whether the molecules satisfy the energy criterion for singlet fission (*E*_S1_ ≥ 2*E*_T_), we calculated the adiabatic excited states of 2P-PDPP, 2P-TDPP, and 2P-SeDPP with time-dependent density functional theory (TDDFT) (Theoretical methods are described in Methods section). The excitation energies are 1.68, 1.49, and 1.44 eV, respectively. As iSEF creates triplets that are located on the pentacenes, we calculated the vertical triplet energies of the TIPS-pentacene fragments including the acetylene unit (end-capped with an H atom). For triplet energy calculations, we used the ΔSCF method. The first triplet state of the TIPS-pentacene fragments lies 0.69 eV above S_0_. With a singlet energy requirement of 1.38 eV for creating two triplets, singlet fission from S_1_ should be feasible in all three molecules and should be fastest for 2P-TDPP and 2P-SeDPP because 2×*E*_T_ and *E*_S1_ are almost identical. Though these calculated values slightly underestimate the measured S_1_ energies and *E*_T_ of TIPS-pentacene^25^, the trends are correctly reproduced. Moreover, we note that iSEF would also be energetically accessible for all three molecules on the basis of the measured S_1_ energies and *E*_T_ of TIPS-pentacene (~0.8 eV) obtained from phosphorescence measurement^25^, particularly given the energetic stabilization of the ^1^TT state relative to 2*E*_T_^21,24,25,33^.

**Fig. 3:**
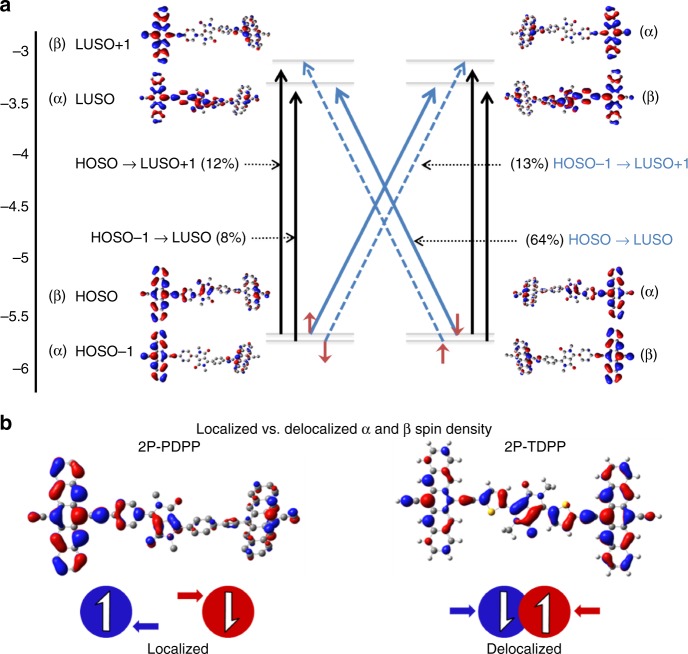
Contributions of orbital replacements and spin density comparison. **a** The electron density change between ground and excited state arises from a linear combination of orbital replacements with CT (blue arrows) and LE (black arrows) character. The HOSO to LUSO transition which contributes strongest (64%), has CR character, moving the α-electron from left to right and the β-electron from right to left. This leads to a partial ^1^TT character of S_1_. **b** α-HOSOs of 2P-PDPP and 2P-TDPP (the symmetry equivalent β-orbitals are omitted). In 2P-PDPP α- and β-electrons are spatially separated because the SOs are polarized towards opposite pentacenes. In 2P-TDPP the SOs of α-and β-electrons are less polarized and involve the bridge more strongly

Analysis of the ground-state wave functions reveals that all three molecules have biradicaloid ground states, i.e., they have significant resonance contributions from biradical structures. Therefore, we focus specifically on the spin properties of the excited states of 2P-PDPP, 2P-TDPP, and 2P-SeDPP and describe them in terms of the spin orbitals (SOs) for α- and β-electrons. As such, α and β-electrons have the same energy but reside in SOs that are symmetry equivalents and tend to be localized on opposite ends of the molecules. In Fig. 3a the frontier SOs of 2P-PDPP are plotted in order of increasing energy. The highest occupied spin orbitals (HOSO) and HOSO–1 and the lowest unoccupied spin orbitals (LUSO) and LUSO+1 are localized mostly on pentacene. HOSO–1 and HOSO as well as LUSO and LUSO+1 are very close in energy. The S_1_ state does not arise from a single HOSO-LUSO transition but rather, like all excitations with energies up to 520 nm, from a linear combination of transitions involving HOSO, HOSO–1, LUSO, and LUSO+1. As in closed-shell calculations, α- and β-electrons contribute equally.

For 2P-PDPP the largest contribution to S_1_ (64%) comes from the HOSO-LUSO transitions. From Fig. 3, it is clear that due to the localized nature of α and β SOs on opposite pentacenes, the HOSO-LUSO transitions of α- and β-electrons individually have charge-transfer (CT) character and their superposition creates a charge resonance (CR) state. The net effect of the CR process is to exchange the electron spins between the pentacenes without creating any charge separation. If the HOSO-LUSO CR transitions were the only contributions to S_1_, 2P-PDPP would be in the ^1^TT state directly after photoexcitation. However, there are additional contributions to S_1_: HOSO–1 to LUSO+1 (13%), (a CR state in reverse direction compared to HOSO-LUSO), plus local excitations (LE) HOSO to LUSO+1 (12%), and HOSO–1 to LUSO (8%). The LE contributions to S_1_ are essential because the large spatial overlap between HOSO and LUSO+1 and between HOSO–1 and LUSO contributes oscillator strength to S_1_.

Taking all the contributions together and considering that HOSO to LUSO and HOSO–1 to LUSO+1 CR states move the electrons in opposite directions, the strongly allowed S_1_ state has 77% CR and at least 50% ^1^TT character. The electron density on the bridge increases by 0.14 eV during excitation to S_1_. Indeed, the PDPP bridge is crucial in bringing about the coupling between LE and CR states, a finding which is independent of the density functional used (global hybrid or range-separated, Supplementary Figure 17). We therefore conclude that S_1_ is a superposition of LE and CR states resulting in a partial ^1^TT character of the bright S_1_ state.

## Discussion

Based on the ultrafast triplet formation kinetics in 2P-PDPP, we invoke iSEF as the generation pathway. This can also be inferred from the subsequent triplet decay within hundreds of picoseconds (213 ps), which can only be rationalized through intramolecular triplet-triplet annihilation. In a dimer such as 2P-PDPP the triplets generated by iSEF will reside on the two pentacene units, separated by the PDPP bridge. In pentacene dimers reported earlier, it has been demonstrated that the absorption signatures of the coupled triplet-pair states ^1^TT cannot be distinguished from free or uncoupled triplets if the inter-chromophore separation is more than few C–C single bonds^31^. In 2P-PDPP the triplet pair is spatially separated by the length scale of the π–PDPP–π bridge, a distance of ~1.5 nm. At this spacing free triplets and a weakly coupled ^1^TT pair would be spectrally identical. However, the rapid geminate annihilation behavior is most consistent with the coupled pair state^34^, and we thus assign the signature obtained at 1 ps specifically to weakly coupled triplet pairs.

The solvent polarity dependence of singlet fission can be rationalized by invoking a charge resonance (CR) character of the S_1_-state in 2P-PDPP that arises from a superposition of charge transfer processes between the two terminal pentacenes (as described in the Theoretical methods section). In the other two derivatives, the S_1_ states do not have CR character and we observe neither iSEF nor any solvent dependence. This idea was independently tested by mixing two solvents that support iSEF but show different rates. We found that the triplet generation in toluene is slowed down considerably as compared to chlorobenzene. This rate increases when chlorobenzene is titrated with toluene (Supplementary Figure 14) clearly indicating the role of CR states in dictating the iSEF process. This indicates that the PDPP bridge plays a critical role in facilitating the CT coupling in the multi-excitonic singlet state. In fact the significance of charge resonance character in driving iSEF in these pentacene dimers has also been observed recently in covalent terrylenediimide dimers thereby generalizing our observation in other molecular frameworks^35^.

It is important to recognize that although 2P-TDPP and 2P-SeDPP have biradicaloid ground states, neither of them undergoes iSEF. We conjecture that the inhibition of iSEF arises from the electronic nature of the SOs. The α-HOSOs of 2P-PDPP and 2P-TDPP are shown in Fig. 3b. Supplementary Figure 18 compares additional frontier α-SOs of 2P-PDPP with those of 2P-TDPP and 2P-SeDPP (β-SOs are not shown as they are symmetry equivalents of the α-SOs). It is clearly visible that HOSOs and especially LUSOs of 2P-TDPP and 2P-SeDPP are delocalized, having less spin density on pentacene, and stronger contributions from the bridges. Because of this delocalization, HOSO to LUSO transitions, which dominate S_1_ of 2P-TDPP and Se-PDPP with contributions of 87% and 85% respectively, have little CR character and do not exchange spins across the molecules. Hence, unlike in 2P-PDPP, the S_1_ states of 2P-TDPP and 2P-SeDPP do not have ^1^TT character. In order to understand the dependence of spin density localization on the backbone planarity of pentacene-π–DPP–π-pentacene dimeric structures, we carried out electronic structure calculations on artificially distorted 2P-PDPP and 2P-SeDPP in their planar and non-planar structures, respectively. For 2P-PDPP the CR character of S_1_ increases on planarization, while breaking the planarity of 2P-SeDPP results in further localization of the LUSO on the pentacenes (Supplementary Figure 19). Interestingly, distortion from the equilibrium geometry thus appears to favor iSEF in both molecules therefore indicating that our observed functional differences are not a simple function of only planarity.

Vibronic mixing of S_1_ and ^1^TT has been suggested to explain 2D photon echo measurements of pentacene films^36^ and rapid endothermic SEF in TIPS-tetracene^21^. Here, we did not consider any vibrational coupling but find that biradical character is essential as CR states can not occur in closed-shell molecules, where α- and β- electrons occupy identical space orbitals. Compared to CT-mediated coupling of S_1_ and ^1^TT states discussed in the literature^9,10,16,37^, there are three main differences in the present work. Firstly, the CR character occurs directly in S_1_ rather than evolving from S_1_. Moreover, due to the simultaneous CTs in opposite directions, no ion pairs are formed and the energy of the CR state is lower than that of a CT state. Finally, unlike the CT mediated mechanism, which requires further electron transfer to reach ^1^TT, the present mechanism requires no further intermediate states as S_1_ already has partial ^1^TT character and can therefore relax very fast to the lower lying ^1^TT state. We propose that this process can be described adiabatically^8,33^, where relaxation along the S_1_-^1^TT potential energy surface from the Franck-Condon point results in gradual increase of the ^1^TT character of the wave function. We observe triplet excited state absorption signatures in the NIR instantaneously upon photoexcitation, while the triplet signatures at shorter wavelengths grow as the system relaxes into the ^1^TT minimum^38,39^. It should be noted that the triplet pair state formed at picosecond timescale is a relaxed and weakly coupled state, which has an almost “free” triplet-like transient signature. The reason for this unusual behavior is the nature of the PDPP bridge that sufficiently couples the pentacenes but contributes little enough to the frontier SOs to facilitate a partial exchange of α- and β-electrons during photoexcitation. Subtle changes in solvent polarity or of the donor units in the bridge increase delocalization and prevent iSEF.

To establish whether this mechanism is unique to 2P-PDPP, we have investigated other pentacene dimers as reported by Lukman et al.^16^, Sanders et al.^31^, and Zirzlmeier et al.^40^. In addition, we considered modified 2P-PDPP by replacing pentacene with tetracene, hexacene, and heptacene (Supplementary Figure 20). According to our preliminary results, all of the systems with exception of the tetracene analog of 2P-PDPP (molecule 11 in Supplementary Figure 20) have biradical ground states. The nature of the SOs depends strongly on the mode of coupling, the orientation of the pentacenes, and the length of the spacer. When pentacenes are directly coupled and perpendicular to one another (molecules 1 and 2 in Supplementary Figure 20), S_1_ and CT states do not mix. S_1_ is localized on the pentacenes and has low oscillator strength (0.20). Pure CT states with zero oscillator strength are found at about 0.3 eV above S_1_ with the range separated hybrid functional wB97-XD^16^. In end-to-end linked biphenyl (molecule 3 in Supplementary Figure 20) spin densities are only partially localized. S_1_ has CR character but low oscillator strength (0.23). This system was found experimentally to undergo rapid singlet fission but also rapid triplet–triplet annihilation^31^. With phenyl spacers of increasing length (molecules 4, 5 and 10 in Supplementary Figure 20), localization increases and CR character of S_1_ decreases. Only when the pentacenes are coupled to phenyl through acetylene groups^40^ (molecules 6–9, 11, and 12 in Supplementary Figure 20), the oscillator strength of S_1_ gets larger with the following substitution pattern: meta (0.38) < ortho (0.57) < para (0.85) while S_1_ has a strong CR character. With hexacene and heptacene instead of pentacene, spin densities are localized but CR character and oscillator strength of S_1_ are less than with pentacene. This is in line with longer acenes being less efficient SEF chromophores^41^. Thus although most of these systems undergo iSEF, spin density appears to be crucial in controlling iSEF rates, triplet-triplet annihilation, and oscillator strength of S_1_. Among these systems, 2P-PDPP is unique because of its unusually large oscillator strength of S_1_ (1.28). Detailed investigations of the above systems and correlation with experimental data are currently under way in our labs and will be subject to forthcoming publications. Our current work shows that for dimeric pentacene systems with extended π-bridges computing the spin density distribution of the frontier molecular orbitals may turn out to be a critical aspect for rational design of new materials with ultrafast iSEF.

In summary, we have demonstrated here the synthetic control of spin density localization in three pentacene dimers by tuning the electronic nature of the π-bridge, and thereby enabling iSEF. With the optimized PDPP bridge, the partitioning of spin density on opposite sides of the dimer afforded a new coherent spin exchange mechanism, endowing the strongly absorbing S_1_ state with partial ^1^TT character. We have focused here specifically on covalent acene dimers, due to the ease of synthetic tuning and their computational tractability. However, there is no intrinsic barrier to the extension of this design principle and fission mechanism to the more common solid-state systems. We anticipate that spin density localization will prove relevant in those as well, recalling for instance the comparable contribution of equal and opposite charge transfer configurations to the lowest singlet state in crystalline pentacene^37,42^. Indeed, the important role of mixing S_1_, CT and ^1^TT states similar to that revealed by our calculations was recently invoked for heteroacene^21,25^ and zethrene^24^ thin films, pentacene dimers^16^ and terrylene bisimide dimers^41^. It is beyond the scope of our current investigation to determine whether this mixing is accompanied by similar spin density localization effects, but this is an important question for further study that may reveal a more general mechanistic principle. This emergent mechanism should motivate new time-resolved experiments for spin-state tracking in SEF-active materials, while bringing into focus the use of chromophoric bridges, which can simultaneously act as antennas for sensitizing iSEF and fine-tune the properties that enable fission. It has not escaped our attention that the reported coherent spin-exchange mechanism may also be critical in the development of organic materials for light-activated superconductivity where biexciton generation and its transport are functional bottlenecks.

## Methods

### Materials

All the precursor materials used for synthesis of the three pentacene derivatives were obtained from commercial source and used without further purification, Solvents used for were dried and distilled out before being used for the synthesis of the molecules. 2P-PDPP, 2P-TDPP, and 2P-SeDPP were synthesized by two step processes starting from precursors through Sonogashira coupling followed by aromatization. Experimental procedure and synthetic methods are described in Supplementary methods. Intermediates and final compounds were purified by silica gel chromatography; structures and their purity were verified by ^1^H, ^13^C NMR, and MALDI-TOF.

### Steady-state absorption and emission spectroscopy

UV–Vis spectra were recorded on Perkin-Elmer (Lambda 35) UV–Vis Spectrometer. Steady state fluorescence emission studies were carried out with a Spex FluoroLog-3 spectrofluorometer (Jobin- Yvon Inc.).

### Flash-photolysis experiment

Laser Kinetic Spectrometer (Edinburgh Instruments, UK, model LP920) was used for nanosecond flash photolysis experiments at BARC, Mumbai. The sample in a 1 cm × 1 cm cuvette was pumped using a 7 ns laser pulse at 532 nm generated using a frequency doubled Nd-YAG laser (Thales Laser SA, France, model: SAGA). A pulsed 450 W xenon arc lamp generates the white light continuum (300−1000 nm) probe pulses.

### Transient absorption measurements

The detailed description about the home-built pump-probe/transient absorption setup has been described in detail earlier^43^. Briefly, a modelocked Ti:Sapphire laser oscillator (Coherent Micra-5 system) produces the fundamental laser pulse with 100 nm bandwidth, 27 fs pulse width, 80 MHz repetition rate, and 5 nJ/pulse power which was then amplified using a regenerative amplifier (Coherent Legend Elite^®^) to generate amplified pulses with 60 nm bandwidth, 1 kHz repetition rate, 30 fs pulse width and 4mJ/pulse power. Part of this amplified femtosecond pulse was guided through an ultrafast optical parametric amplifier system (Coherent OPeraASolo^®^) to generate tunable pump pulses which were used to excite the molecules of interest. Another part of the fundamental beam was passed through a 2 mm thick sapphire crystal to generate white-light probe continuum. The visible part (420–750 nm) of the broadband probe continuum was separated using a 800 nm short-pass filter while the NIR regimes (850–1300 nm) was selected using a 850 nm long pass filter. The actinic pump and probe pulses were focused and overlapped on the sample inside a flow cuvette with 2 mm glass window. The transmitted probe after the sample was dispersed using Helios^®^ spectrograph onto an imaging element. The temporal resolution for the set up was determined to be near 90 fs. All pump-probe measurements were performed under flowing condition with the liquid driven by a peristaltic pump which ensures fresh sample before every laser shot. Pump pulse energy was attenuated to 100nJ in order to minimize the photo-damage.

### Theoretical methods

Ground and excited states of 2P-PDPP, 2P-TDPP, and 2P-SeDPP were investigated with density functional theory (DFT) and time-dependent DFT (TDDFT). Test calculations with and without the triisopropylsilyl (TIPS) groups show that TIPS groups influence neither singlet-triplet splitting nor excitation energies. Likewise replacing alkane chains with methyl groups does not influence the electronic structure (Supplementary Figure 10). Therefore, the truncated systems were used for most of the calculations. As low lying excited states of large π-systems are not diffuse, the 6-31G* basis set was used throughout. Based on previous experience with conjugated and biradicaloid conjugated systems and further test calculations, the B3P86 functional with 30% of exact exchange was chosen^44,45^. Solvent effects of chlorobenzene and cyclohexane were included with the polarized continuum model (PCM) as implemented in Gaussian 09. All calculations are done with Gaussian 09, Rev. A 02^46^.

To characterize the ground states, closed-shell wave functions were checked for stability. Closed-shell wavefunctions of all three molecules have internal instabilities. At the B3P86-30%/6-31G* level the biradicaloids lie 0.02 eV (2.4 kJ/mol), 0.05 eV (4.8 kJ/mol), and 0.12 eV (11.9 kJ/mol) below the closed-shell forms for 2P-PDPP, 2P-TDPP, and 2P-SeDPP, respectively. This raises the issue of applicability of broken symmetry DFT (BS-DFT) as biradicals have two-determinantal wave functions while DFT uses only a single Slater determinant. Davidson and Clark investigated biradicals and concluded that for large systems for which multi-reference methods cannot be employed, BS-DFT is the best choice of method^47^. Casida showed that excited state calculations with BS-TDDFT are reliable as long as the spin is conserved^48^. The applicability of TDDFT for states relevant to singlet fission of medium-sized biradicaloid systems was scrutinized vs. multi-reference approaches by Grotjahn et al^49^. The study confirms that single reference methods are reliable and that global hybrids are only marginally worse than local hybrids for singlet states. For triplets, the ΔSCF method was shown to yield reliable triplet energies.

Following the above studies, we calculated the triplet energies with the ΔSCF method as energy differences between BS-singlet and triplet ground states. We also confirm the applicability of BS-TDDFT as we observe no spurious or missing states in the BS-TDDFT absorption spectrum 2P-PDPP compared to the closed-shell spectrum (Supplementary Figure 16), although the BS ground state has an expectation value of the spin operator of 0.95.

Analysis of the S_1_ state of 2P-PDPP reveals a significant amount of charge transfer (CT) character. This touches another issue of TDDFT, the known underestimation of the energy of charge transfer states^50^. To assure that the CT admixture to S_1_ is not an artifact, we compared absorption spectra of 2P-PDDP and of two different fragments obtained from 2P-PDPP by removing first the DPP bridge (fragment 2) and second also the phenyl groups (fragment 1) without reoptimizing the rest of the structure. This produces separate locally excited (LE) and CT states for the fragments. SI Figure S11 shows structures, excitation energies, and calculated spectra. Excited state calculations in the gas phase for the fragments with the B3P86-30% and the wB97X-D functionals predict LE states at 1.91 and 1.83 eV with B3P86-30% and at 2.21 and 2.22 eV with wB97X-D. These LE states have exactly the same energies as the corresponding excitations of the individual molecules. The fragments, however, have additional CT states at 2.49 eV (fragment 1) and 2.30 eV (fragment 2) with B3P86-30% and at 4.64 and 4.38 eV, respectively with wB97X-D. The wB97X-D CT states match the energy difference between ionization potential (IP) and electron affinity (EA) of 4.72 eV closely but the B3P86-30% CT excitation energies are lower than IP-EA of 3.75 eV, as expected. Although wB97X-D predicts CT states correctly at much higher in energy, the S_1_ state of 2P-PDPP is composed of a linear combination of LE and CT excitations with both functionals. S_1_ is predicted at 1.68 eV (B3P86-30%) and at 2.10 eV (wB97X-D). Such increases in energy are observed generally with range-separated funstionals^1^. With both functionals no pure CT states similar to those of the fragments are obtained. The admixture of CT excitations to S_1_ is therefore not an artifact of the B3P86-30% functional. This indicates that incorporation of the DPP bridge changes the character of the S_1_ state, leading to a red shift and a large increase in oscillator strengths. Comparing the B3P86-30% and wB97X-D spectra with the experimental one, reveals that the B3P86-30% absorption spectrum is in much better agreement with experiment in terms of peak positions, peak spacings, and relative oscillator strengths. Therefore, the B3P86-30% functional is used hereafter.

## Supplementary information

Supplementary Information

## Data Availability

The data sets generated and analyzed during the current study are available from the corresponding authors on reasonable request.
