# Supplementary material for: Spin density encodes intramolecular singlet exciton fission in pentacene dimers
Source: Nat Commun. 2019 Jan 3;10:33. doi: 10.1038/s41467-018-07736-3 (PMC6318326; doi:10.1038/s41467-018-07736-3)
Supplement: Supplementary file 1 — Supplementary Information [file 41467_2018_7736_MOESM1_ESM.pdf]

SUPPLEMENTARY INFORMATION

Spin Density Encodes Intramolecular Singlet Exciton Fission in Pentacene Dimers

KC Krishnapriya *et al.*

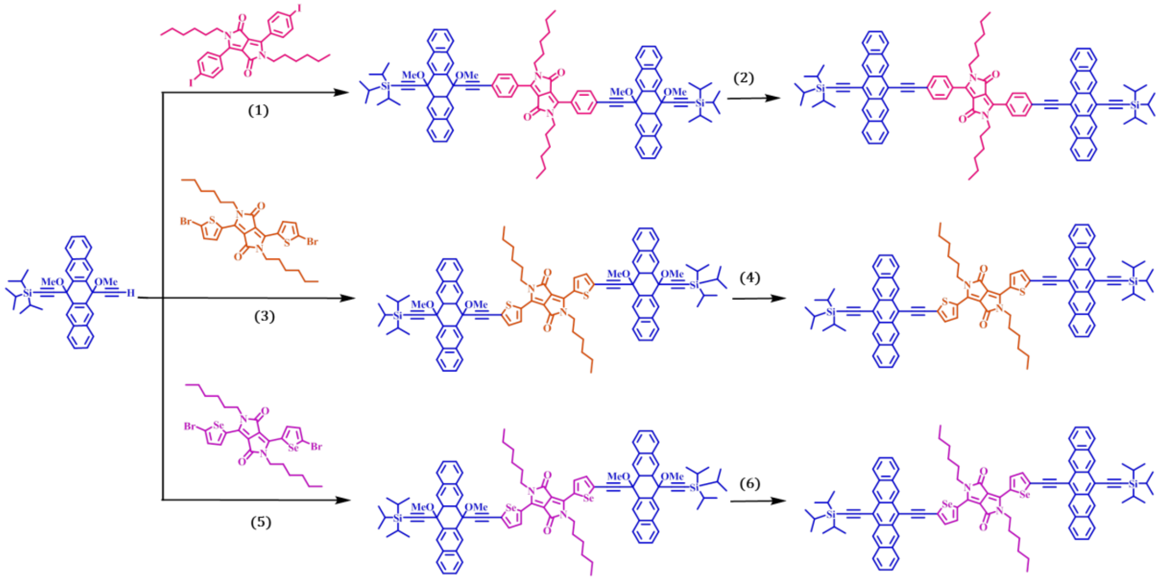

(1) $Pd(PPh_3)_4$, CuI, THF/DIPA, 45 °C, 24h, 80%. (2) $SnCl_2.2H_2O$, THF, rt, 6h, 70%. (3) $Pd(PPh_3)_4$, CuI, THF/DIPA, 45 °C, 24h, 75%. (4) $SnCl_2.2H_2O$, THF, rt, 6h, 60%. (5) $Pd(PPh_3)_4$, CuI, THF/DIPA, 45 °C, 24h, 75%. (6) $SnCl_2.2H_2O$, THF, rt, 6h, 60%.

Supplementary Figure 1. The synthetic scheme for 2P-PDPP, 2P-TDPP and 2P-SeDPP by two step processes starting from precursors through Sonogashira coupling followed by aromatization. The synthetic route for the target molecules 2P-PDPP, 2T-DPPP and 2P-SeDPP is shown in Supplementary Figure S1. Monomers were synthesized according to literature procedures.[1,2] The target compounds were obtained by two step processes, starting with Sonogashira coupling of pentacene (P) to the diketopyrrolopyrrole (DPP) derivatives using $Pd(PPh_3)_4$ as the catalyst. The synthesis of the target compounds was achieved by aromatization of the intermediates in the presence of $SnCl_2.2H_2O$. Intermediates and final compounds were purified by silica gel chromatography; structures and their purity were verified by ^1H, ^{13}C NMR, and MALDI-TOF.

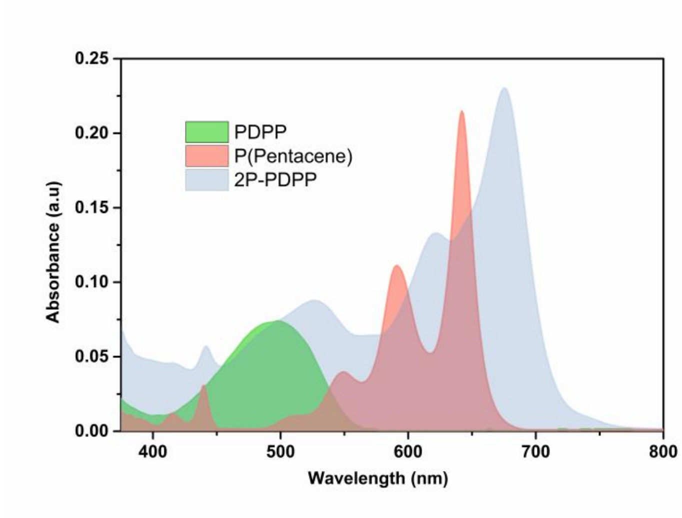

Supplementary Figure 2. Comparison of absorption spectra of 2P-PDPP with Pentacene and PDPP
Comparison of absorption spectra of Phenyl DPP and Pentacene with 2P-PDPP in Chlorobenzene. From the Supplementary Figure the absorption features of 2P-PDPP can be identified with contributions from both PDPP and Pentacene.

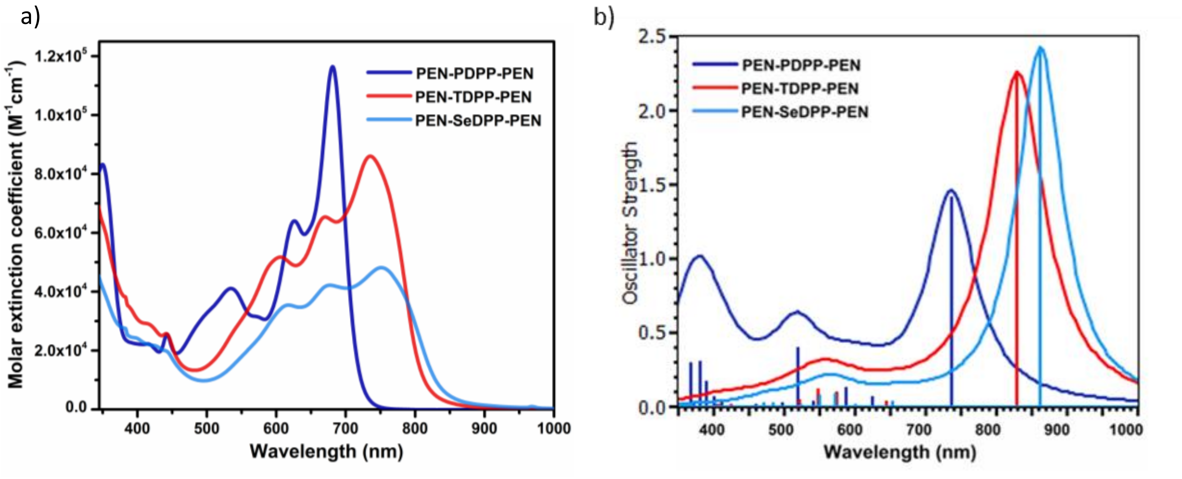

Supplementary Figure 3. Absorption spectra of 2P-XDPP dimers in chlorobenzene. Comparison of (a) experimental and (b) computationally calculated absorption spectra of all the three dimers in Chlorobenzene. The UV-Visible absorption spectra of the three dimers are found computationally using UTD-B3P86-30%/6-31G% calculations with maximum error 0.20 eV. As vibrational bands were not calculated, all oscillator strength of the low energy absorptions is found in the single peaks at 738, 830, and 863 nm.

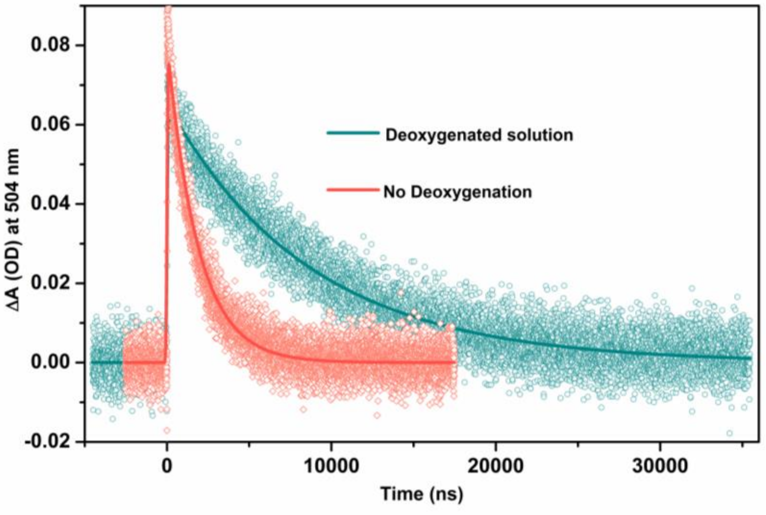

Supplementary Figure 4. nano second Flash photolysis of 2P-PDPP. Comparison of single wavelength kinetics at 504 nm obtained using flash photolysis measurements of 2P-PDPP solution in chlorobenzene. The red kinetics was obtained under O_2 atmosphere and yields a lifetime of 1865.9 ns. The green kinetics, the data recorded after deoxygenation with similar experimental conditions yield a decay time of 8655.3 ns. On the basis of this oxygen sensitivity, the species absorbing here can be assigned as the 2P-PDPP triplet.

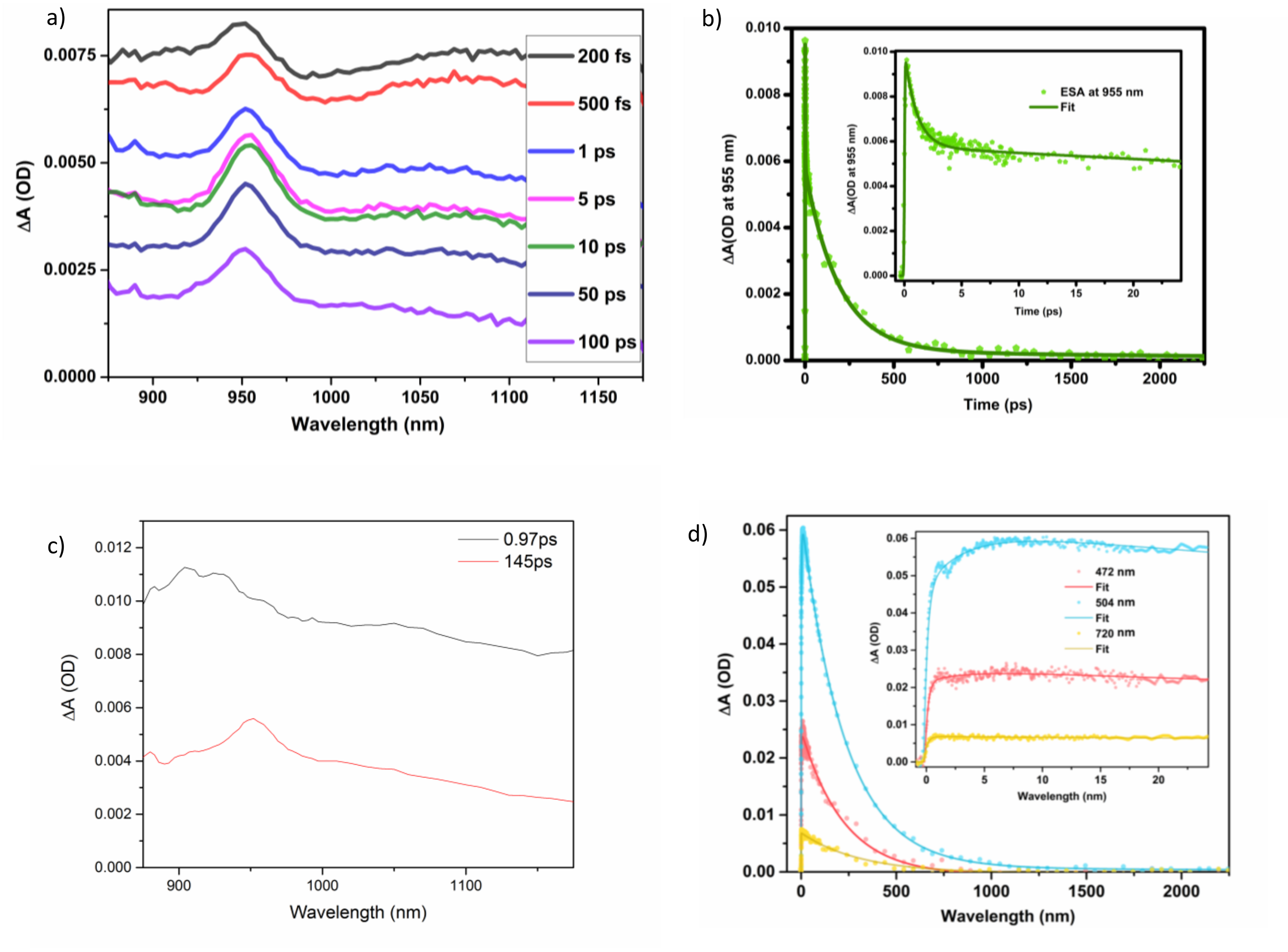

Supplementary Figure 5. fs Transient absorption spectra and kinetics of 2P-PDPP in the NIR region. Transient absorption (a) spectra and (b) kinetics at 955 nm with exponential fit of 25 μM solution of 2P-PDPP in chlorobenzene using 670 nm as pump pulse and Near-IR probe pulse (875-1175nm). c) Global analysis of the TA spectra in the NIR region showing two components with decay time constants of 0.97 and 145 ps. d) TA kinetics of 2P-PDPP at 472, 504 and 720 nm.

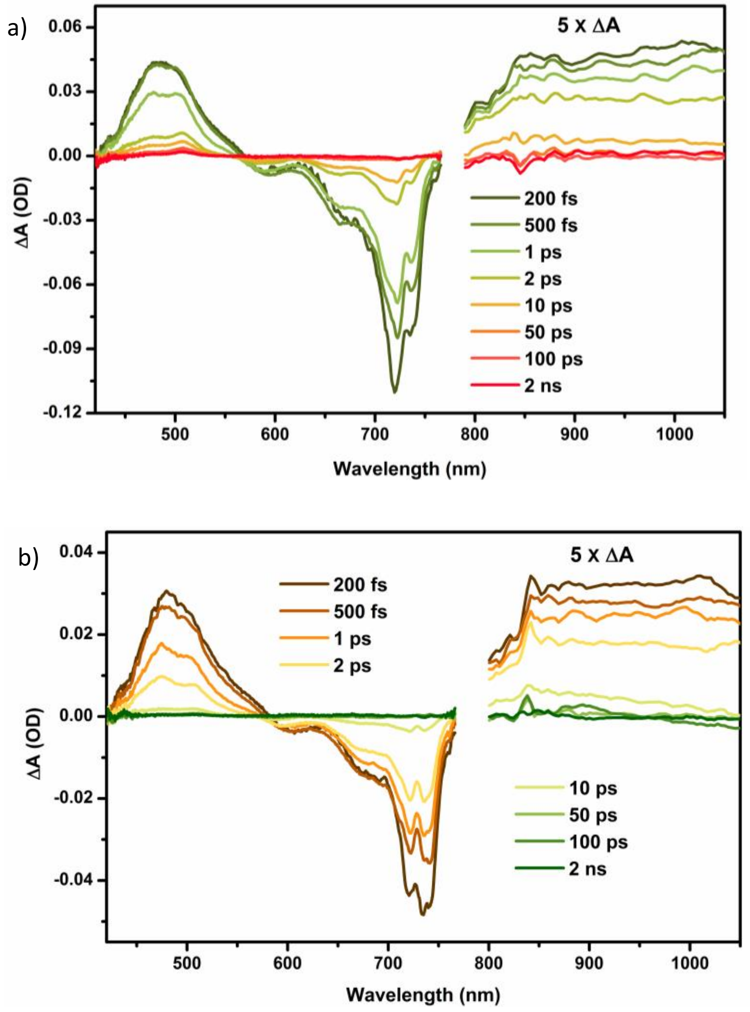

Supplementary Figure 6. fs Transient absorption spectra of 2P-TDPP and 2P-SeDPP. fs Transient absorption spectra of 25 μM of a) 2P-TDPP and b) 2P-SeDPP in chlorobenzene using 650 nm as pump pulse. Spectra in the NIR region have been increased in intensity by 5.

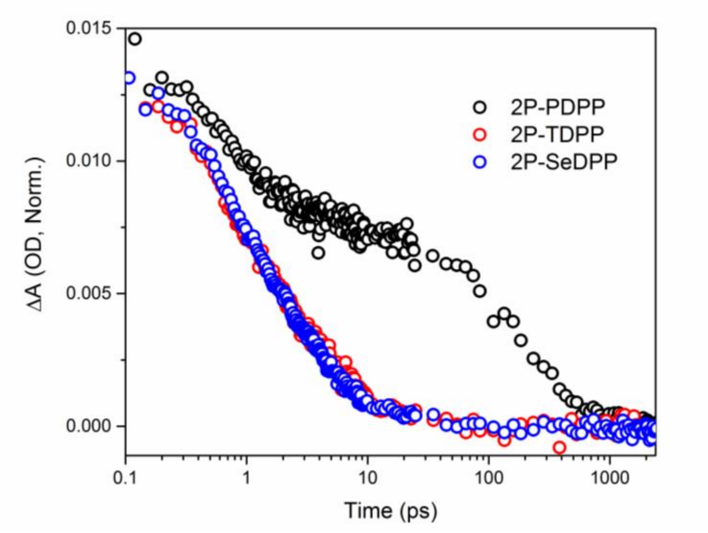

Supplementary Figure 7. ESA kinetics comparison of 2P-XDPP in the NIR region. Comparison of TA kinetics at 955 nm for 2P-PDPP, 2P-TDPP and 2P-SeDPP in chlorobenzene.

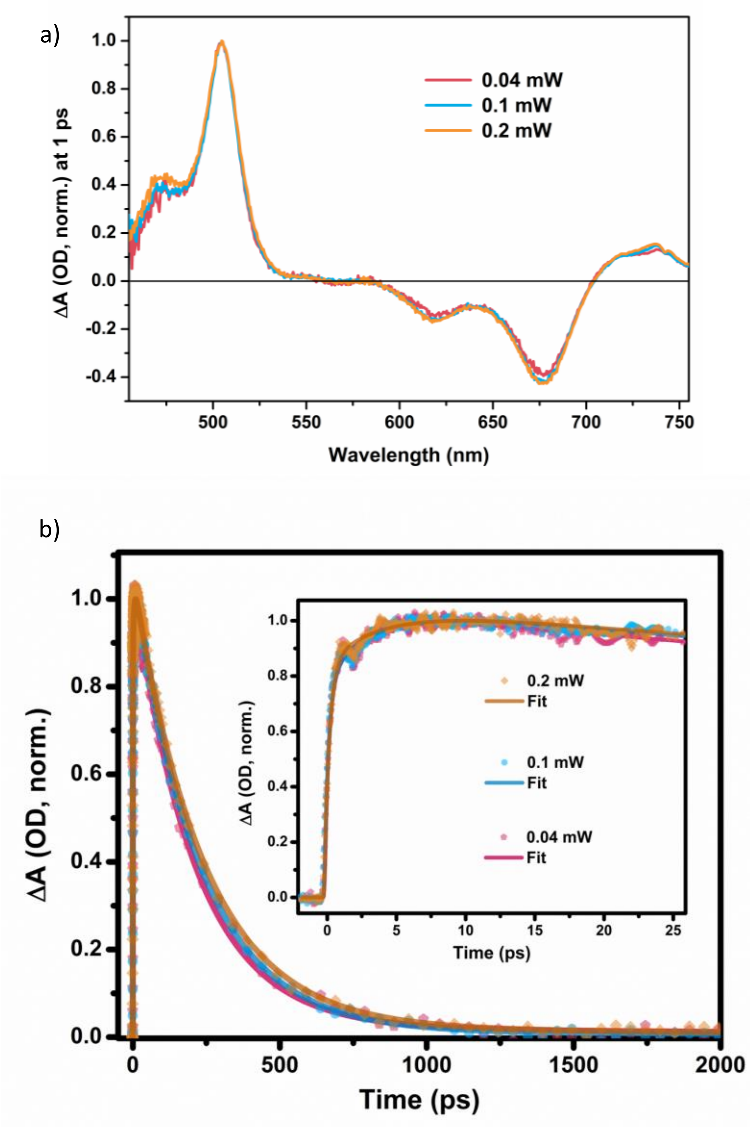

Supplementary Figure 8. Pump power controlled fs transient absorption spectra. a) Comparison of fs TA spectra of 2P-PDPP at 1 ps using different pump powers: 0.04, 0.1 and 0.2 mW and b) Their corresponding single wavelength kinetics at 504 nm with multiexponential fitting, all with the similar time constants (3.1 ps rise and 223 ps decay). Inset: early-time kinetics, when iSEF occurs. fs TA spectra of 25 μM 2P-PDPP in chlorobenzene were collected following excitation at 670 nm under different pump power. TA signals scale linearly with pump power, and the kinetics are found not to vary, confirming that all the measurements are within the linear regime.

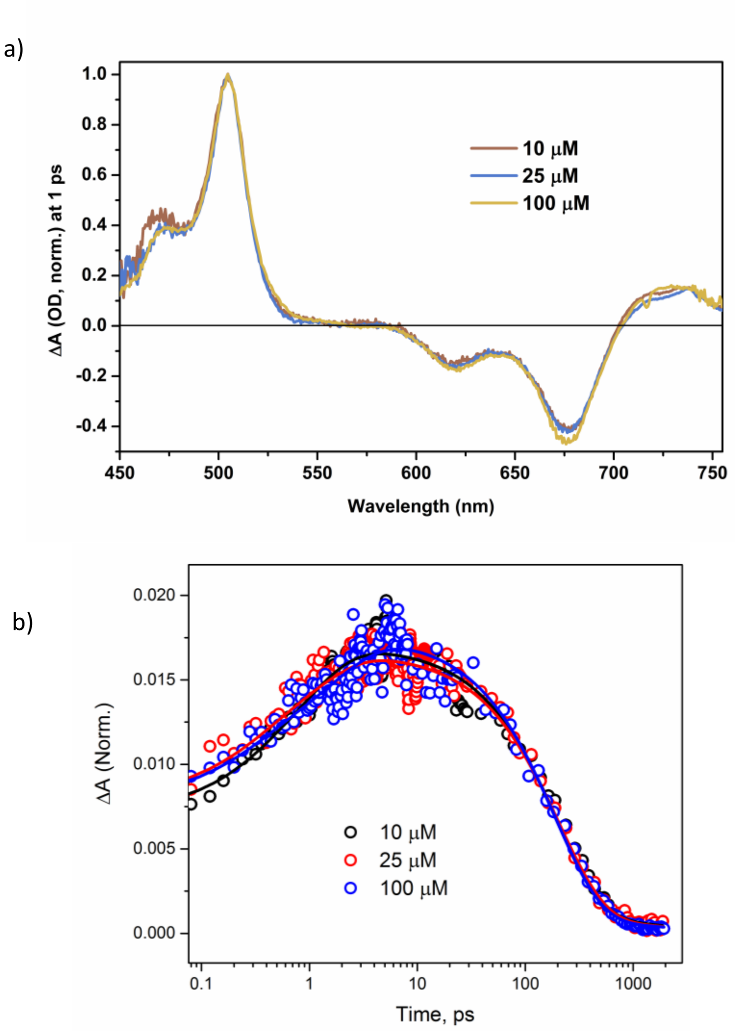

Supplementary Figure 9. Concentration dependant fs-transient absorption spectra. Concentration dependent transient absorption (a) spectra at 1 ps and (b) kinetics of 2P-PDPP collected at 504 nm. All kinetics can be fitted with the similar time constants of 2.6 ps rise and 227 ps decay. fs TA spectra of 2P-PDPP solutions in chlorobenzene were collected over the concentration range 10-100 μM, using excitation at 0.1 mW of 670 nm. Kinetic fitting reveals no change in the excited-state dynamics, confirming that the SEF process observed is entirely intramolecular.

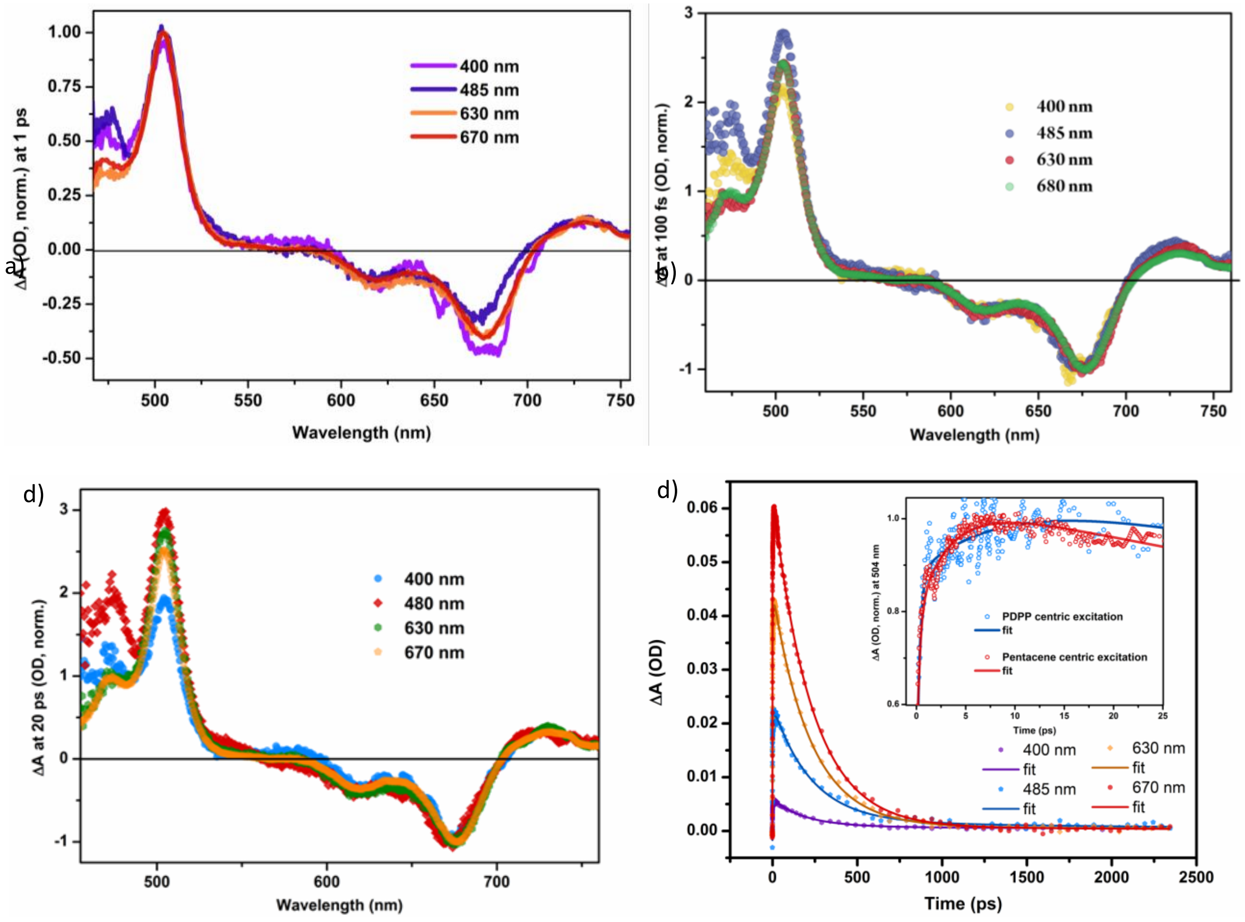

Supplementary Figure 10. Pump energy controlled fs-transient absorption spectra. Comparison of pump wavelength dependent a) 1 ps, b) 100fs and c) 20 ps; (d) the respective kinetics at 504 nm. Kinetics are fitted with multiexponential functions, with rise time constants ranging from 8.6 ps (PDPP centric excitation: 400, 485 nm) to 3.2 ps (Pentacene centric excitation630, 670 nm) and decay time constants ranging from 218ps (PDPP centric excitation) to 221 ps (Pentacene centric excitation). Inset: Early time kinetics with fitting. fs TA spectra were acquired on a 25 μM solution of 2P-PDPP in chlorobenzene following excitation at four different pump wavelengths (of same power 0.1 mW): 400, 485, 630, 670 nm. The same features of iSEF were observed in all cases, but the kinetics revealed a distinct trend of the iSEF rate following higher-energy excitation

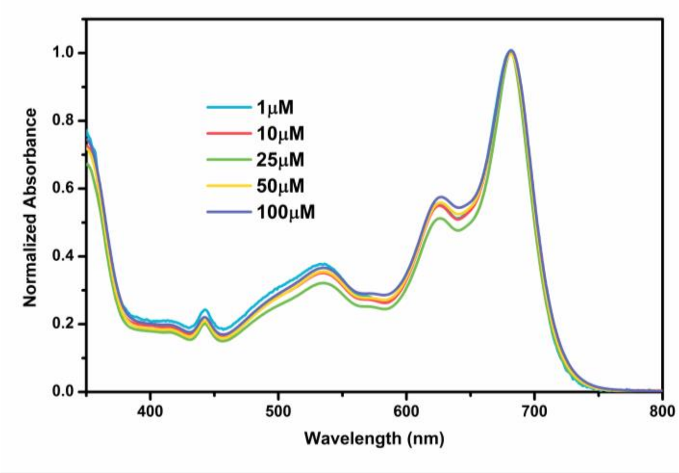

Supplementary Figure 11. Concentration dependent absorption spectra of 2P-PDPP . UV-Vis absorption spectra of 2P-PDPP in chlorobenzene at varying concentrations from 1μM to 100μM, normalized to the low-energy peak.

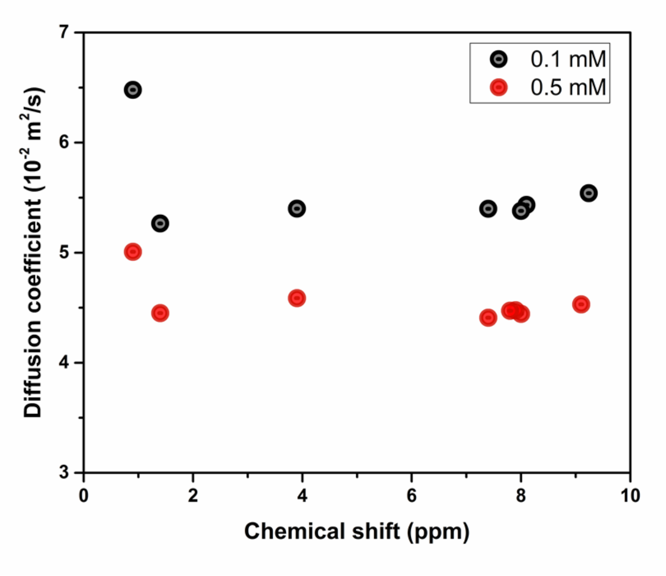

Supplementary Figure 12: DOSY NMR of 2P-PDPP. DOSY-NMR of 2P-PDPP in chlorobenzene at varying concentrations showing the diffusion coefficient values. The similar values are consistent with isolated dimers, rather than aggregates.

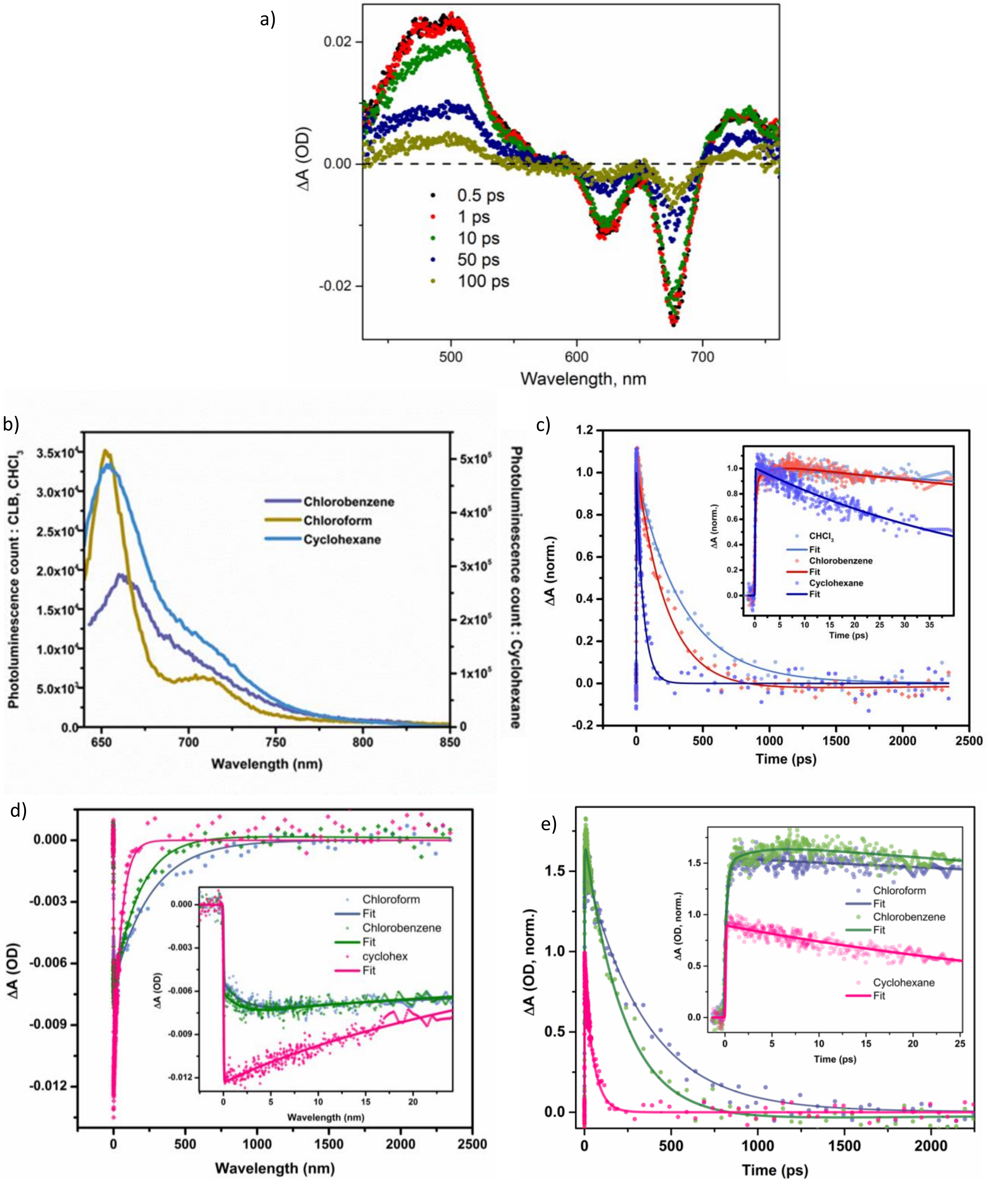

Supplementary Figure 13. Excited state dynamics of 2P-PDPP in different solvents. (a) TA of 25μM PENPDPP in cyclohexane with 0.1mW of 670 nm pump excitation, at different pump-probe delays. The

nonappearance of sharp triplet features near 504 nm indicates that there is no SF in cyclohexane. (b) Steady state photoluminescence spectra of 2P-PDPP of same concentration in three different solvents. Note the spectrum in cyclohexane is on a much larger scale, because iSEF is suppressed in that environment and we observe longer-lived S_1. (c) Normalized TA kinetics of 2P-PDPP in different solvents following photoexcitation at 670 nm. Probe wavelength for all kinetics is 504 nm. Kinetics are fitted with multiexponential functions with time constants (55ps decay in cyclohexane; 3.1ps rise and 227 ps decay in chlorobenzene; or 3.5 ps rise and 316 ps decay chloroform). (d) Raw TA kinetics of 2P-PDPP in different solvents, taken at the ground state bleach peak at 630 nm. The difference in shape on the earliest timescales reveals that the iSEF process occurs over ~3 ps in polar solvents does not occur in cyclohexane. (e) Raw TA kinetics of 2P-PDPP at 472 nm in solvents of different polarity. Insets show detail of early-time dynamics in all kinetic panels.

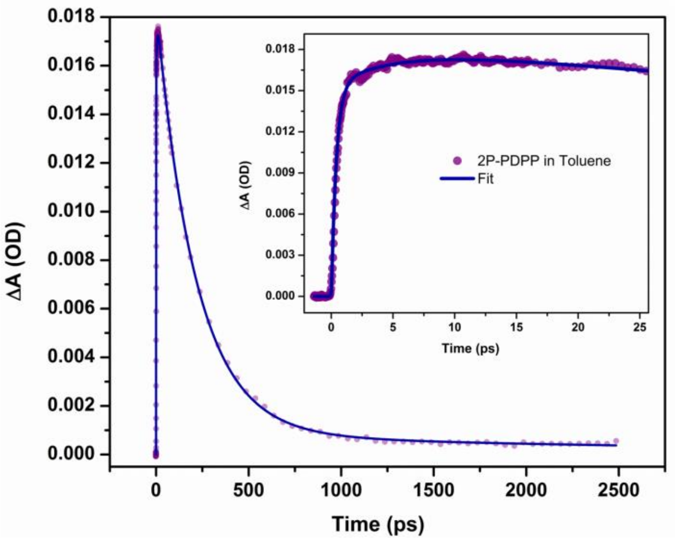

Supplementary Figure 14. Excited state dynamics of 2P-PDPP in Toluene. Solvent dependent singlet fission dynamics studied in toluene, a solvent with intermediate polarity compared to cyclohexane and chlorobenzene. Kinetics of at 509 nm, corresponding to the characteristic sharp triplet feature observed, shows 4.8 ps rise and 213 ps decay similar that observed in chloroform and chlorobenzene. The increased rise time as compared to the higher polarity solvents also supports the proposed charge resonance assisted singlet fission.

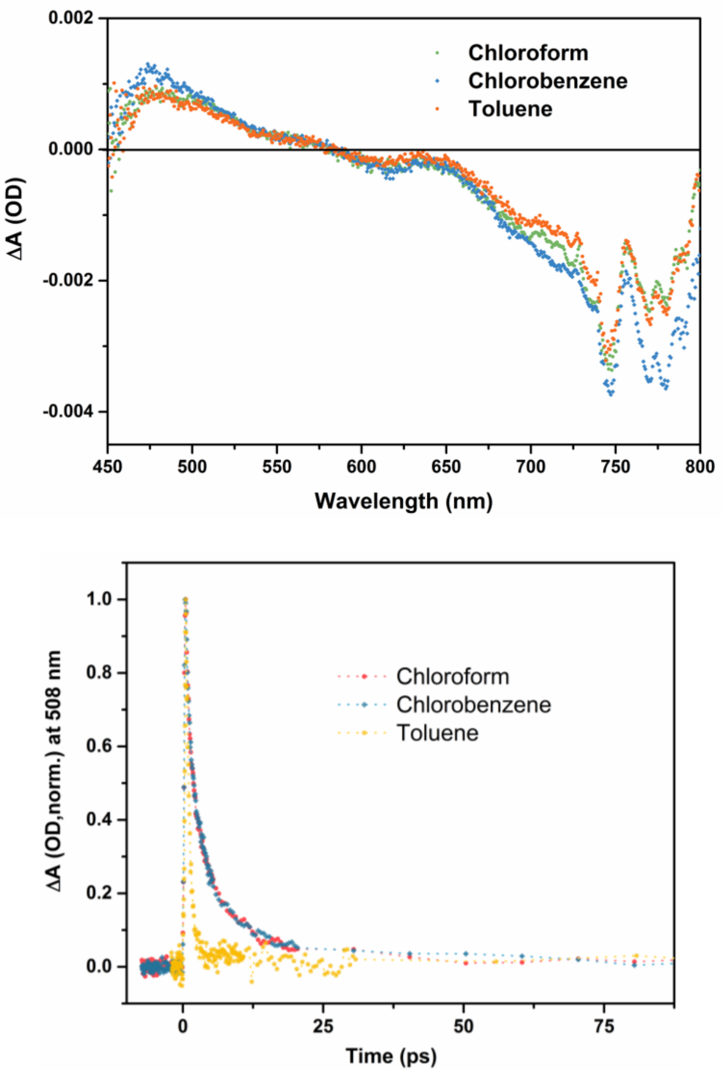

Supplementary Figure 15. Spectra and excited state dynamics of 2P-SeDPP in different solvents. (a) Comparison of transient absorption spectra of 2P-SeDPP in different solvents collected at 1 ps time delay following photoexcitation with 650 nm, 0.1 mW pump. We don't observe any sharp features like that of triplet absorption in the excited state absorption around 500 nm. The excited state absorption feature around 450-550 nm is more similar to ESA of 2P-PDPP singlet state absorption. (b) Kinetics of excited state absorption feature at 509 nm for 2P-SeDPP in different solvents. The kinetics decays within 10 ps.

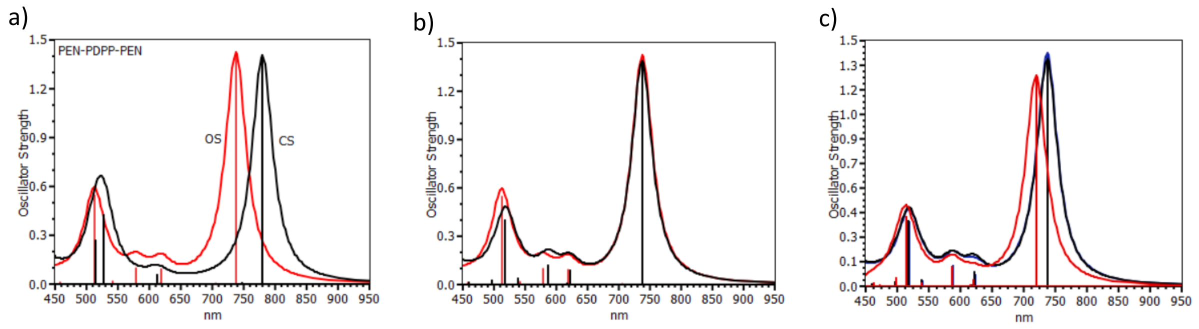

Supplementary Figure 16. Theoretical test calculations for 2P-PDPP. Test calculations for 2P-PDPP at the B3P86-30%/6-31G* level (a) Closed- (CS) vs open-shell (OS) UV-vis spectra in chlorobenzene, (b) UV-vis spectra of complete systems (red) and after removing TIPS and alkyl groups (black) in chlorobenzene, (c) solvent effects gas phase (red), chlorobenzene (black), cyclohexane (blue).

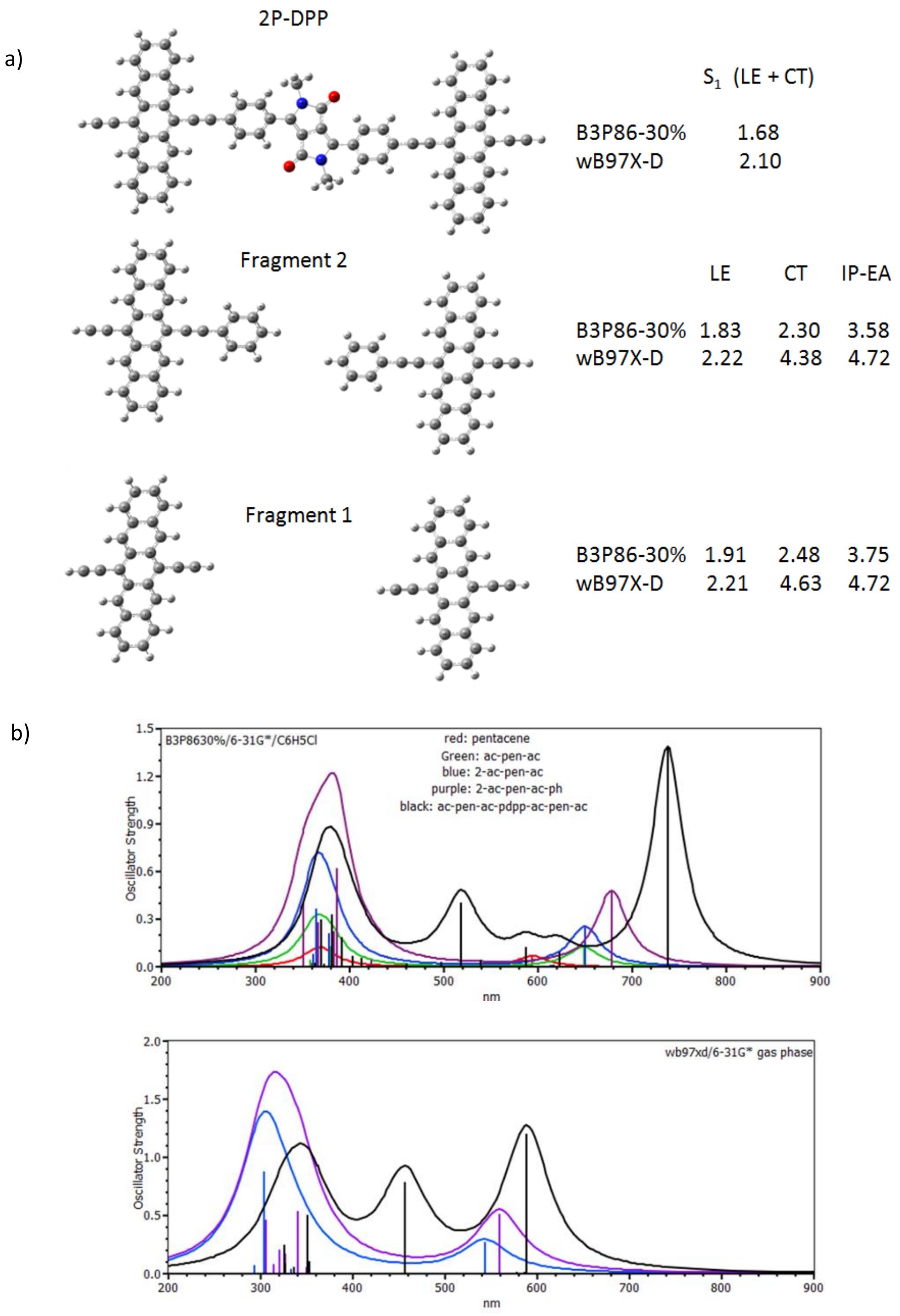

Supplementary Figure 17. Theoretical absorption spectra comparison. Comparison between absorption spectra of 2P-PDPP and fragments without the DPP and PDPP bridges. (a) LE and CT energies with B3P86-30% and wB97X-D functionals. (b) Predicted absorption spectra: Red pentacene, green C_2H_2-pentacene-C_2H_2, blue Fragment 1, purple Fragment 2, black 2P-PDPP.

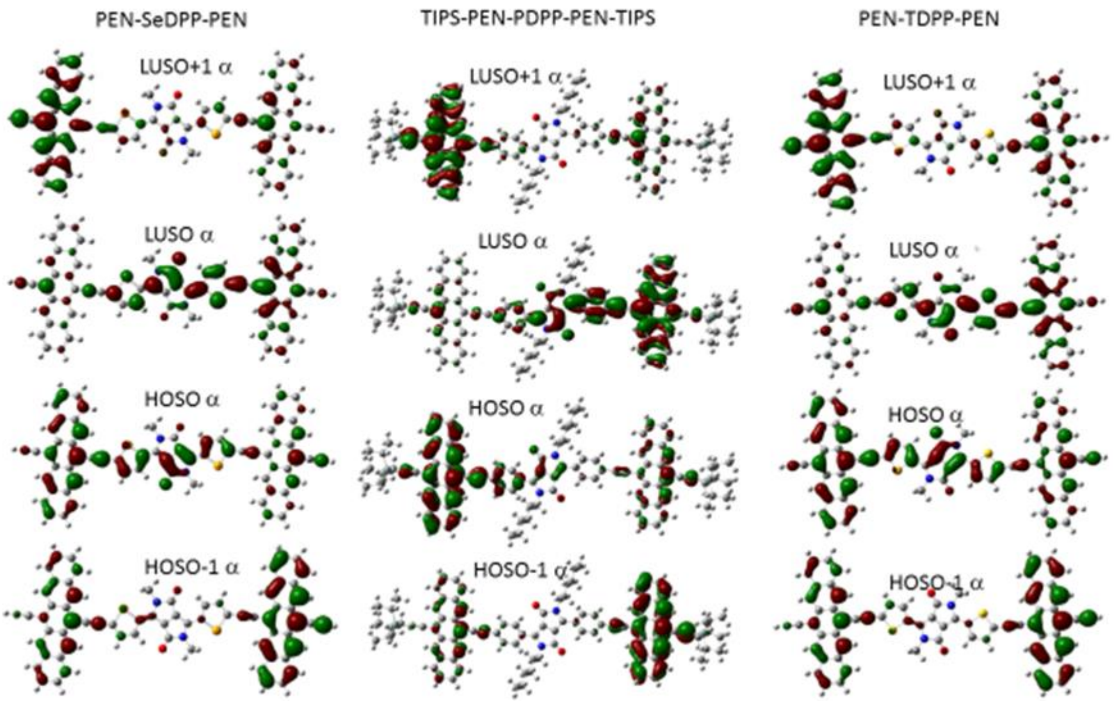

Supplementary Figure 18. Frontier α molecular orbitals of all the three dimers. Frontier α molecular orbitals of 2P-XDPP systems. The four α MOs of importance viz. HOSO-1, HOSO, LUSO, LUSO+1 of all the three 2P-XDPP compounds are shown. Their respective spatial distribution shows that the spin density of α and β spins are localized on the opposite pentacene arms for 2P-PDPP. But for both 2P-TDPP and 2P-SeDPP the spin density is distributed over the entire molecule with substantial spin density on the DPP bridge.

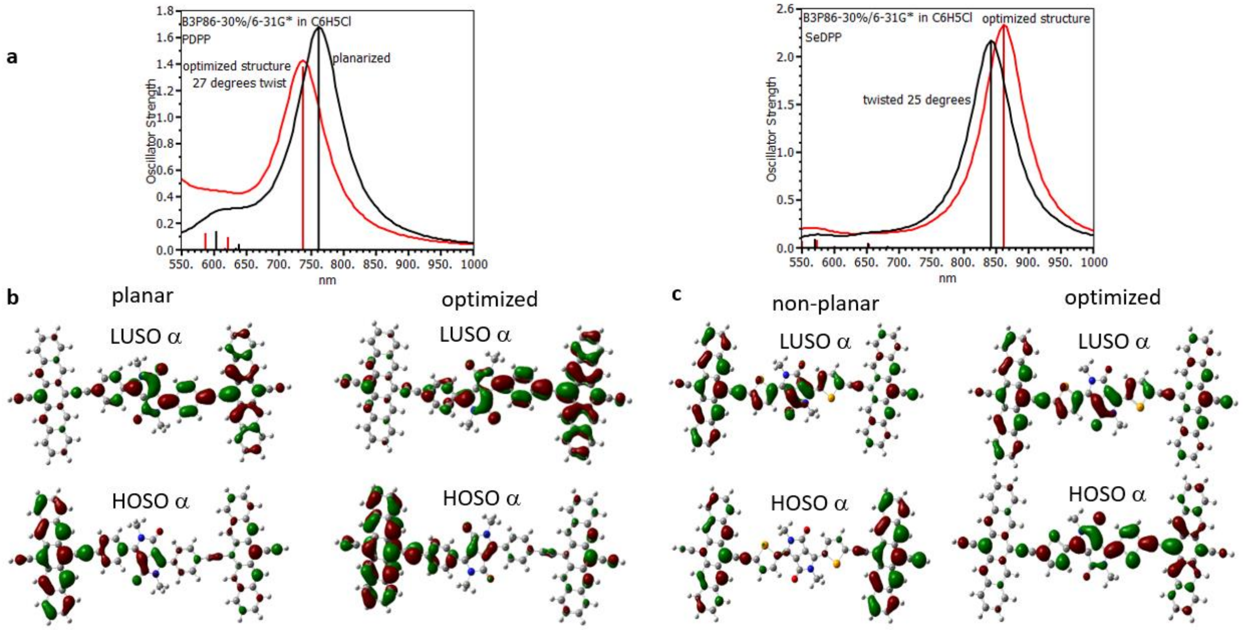

Supplementary Figure 19. Effect on spin density with change in Planarity of 2P-PDPP and 2P-SeDPP. (a) Absorption spectra of optimized and constrained structures of 2P-PDPP and 2P-SeDPP, respectively. Planar forms have larger oscillator strength and peaks are red-shifted by 53 meV, while the difference in absorption peaks between 2P-PDPP and 2P-SeDPP is 242 meV. (b) α spin orbitals of 2P-PDPP in the optimized geometry a twist angle between DPP and phenyl rings of 27 degrees and with a planar structure. (c) α spin orbitals of 2P-SeDPP in the optimized planar structure and with a twist angle between DPP and phenyl rings of 25 degrees.

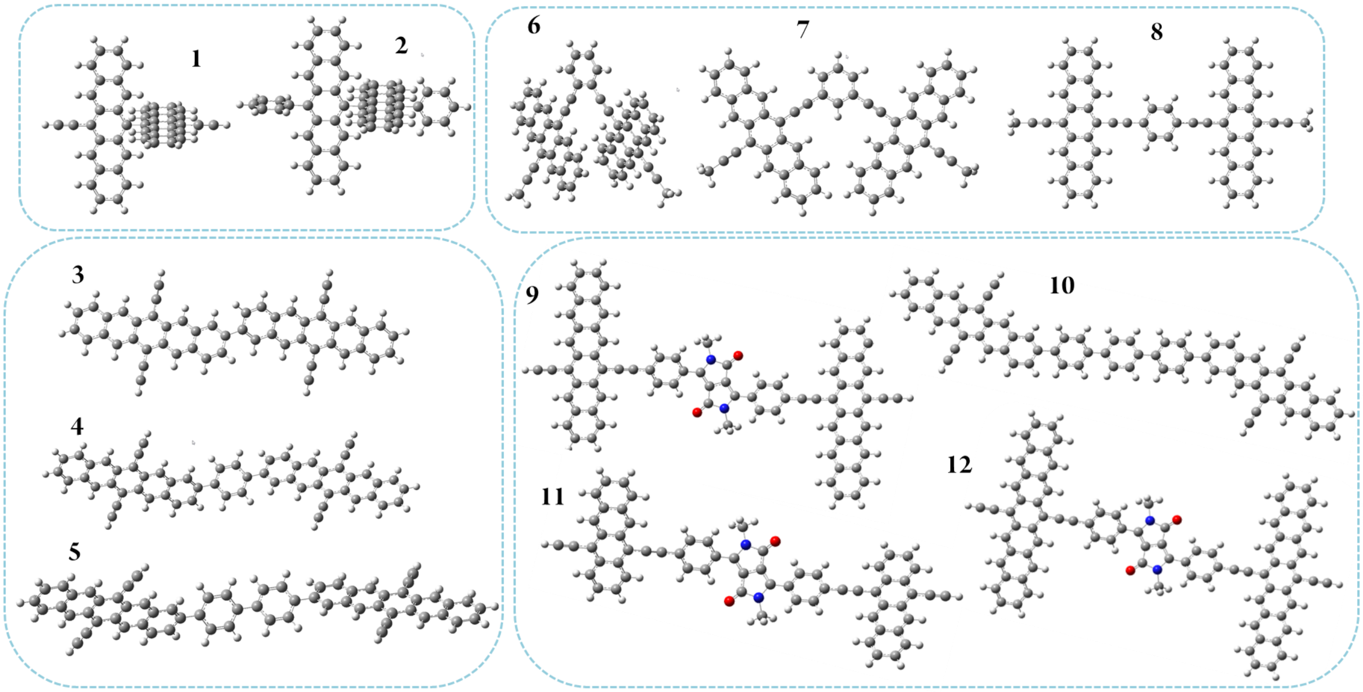

Supplementary Figure 20. Pentacene dimers reported in the literature and newly studied: Orthogonal Pentacene dimers 1-2,[3] End-to-end coupled pentacene dimers 3-5,[4] Regioisomeric pentacene dimers 6-8,[5] Pentacene dimers used in the present contribution 9-12. All the above mentioned molecules were were studied theoretically and the mechanism proposed in this contribution

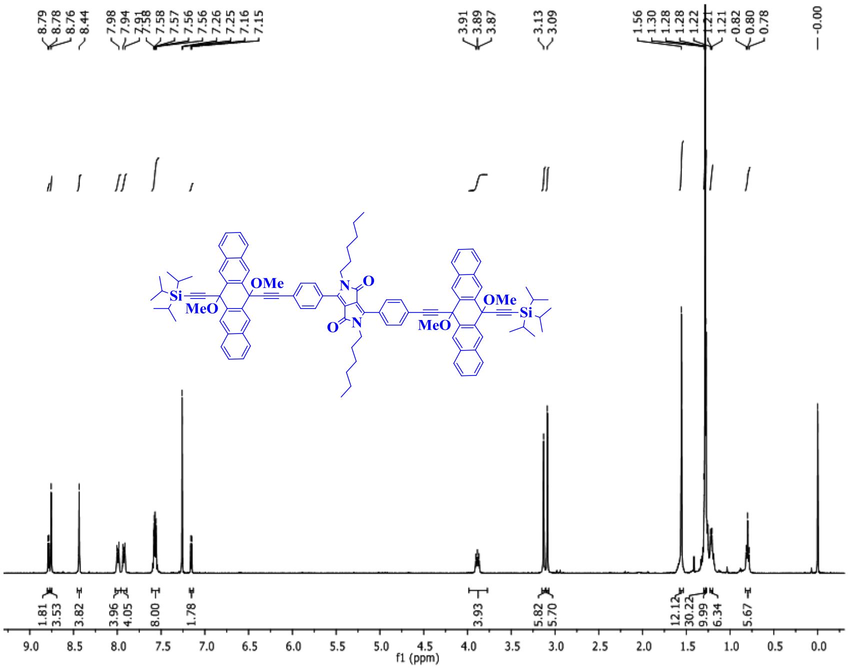

Supplementary Figure 21. ^{1}H NMR ($CDCl_3$, 400 MHz) of (OMe)2P-PDPP(OMe)

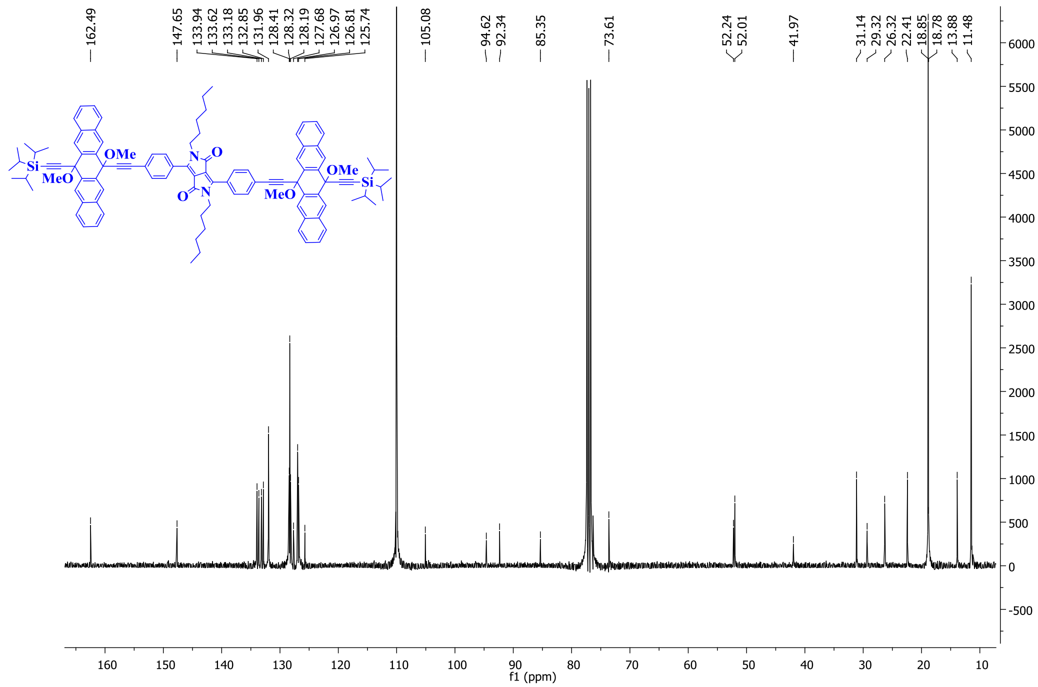

Supplementary Figure 22. ^{13}C NMR ($CDCl_3$, 125 MHz) of (OMe)2P-PDPP(OMe)

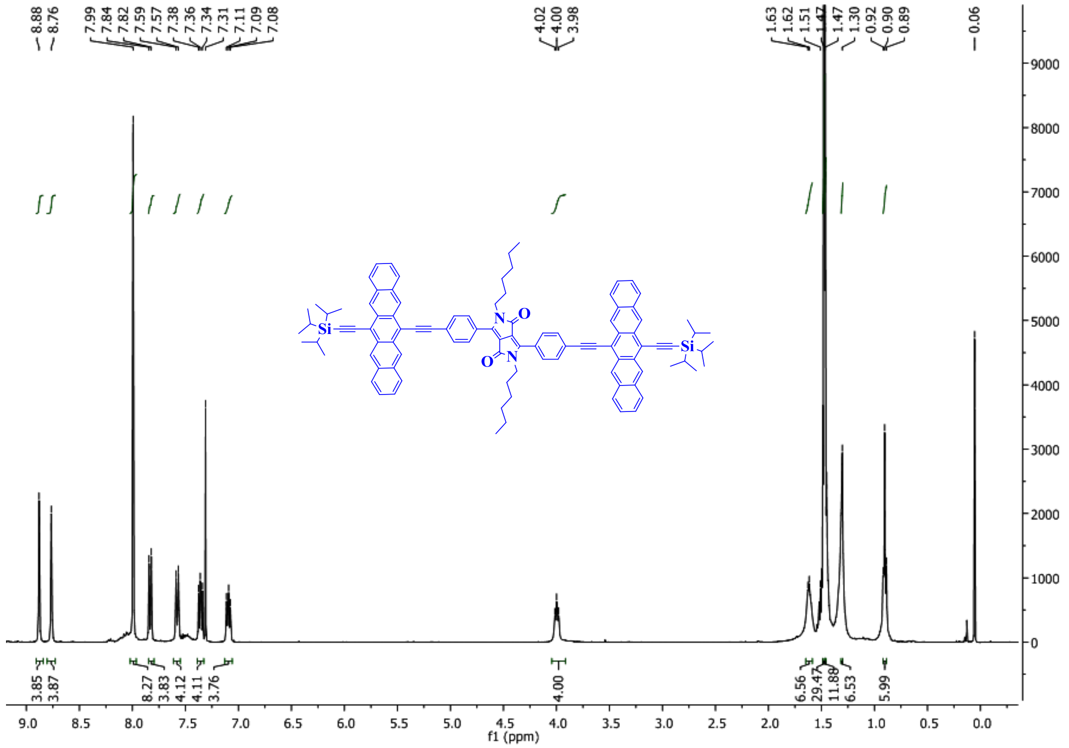

Supplementary Figure 23. ^{1}H NMR ($CDCl_3$, 400 MHz) of 2P-PDPP

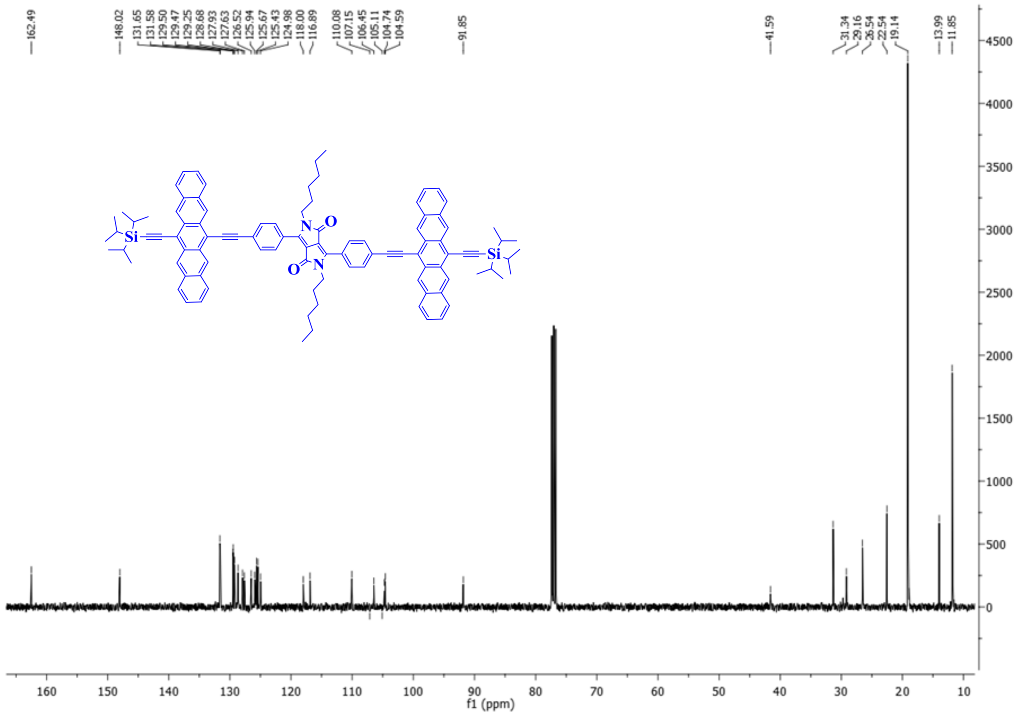

Supplementary Figure 24. ^{13}C NMR (125 MHz, $CDCl_3$) of 2P-PDPP

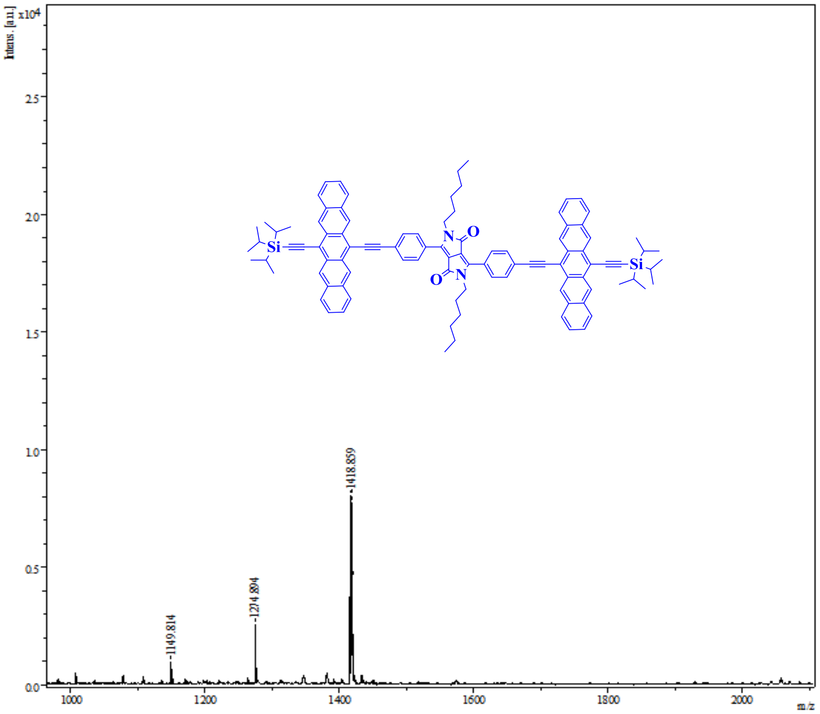

Supplementary Figure 25. MALDI-MS of 2P-PDPP

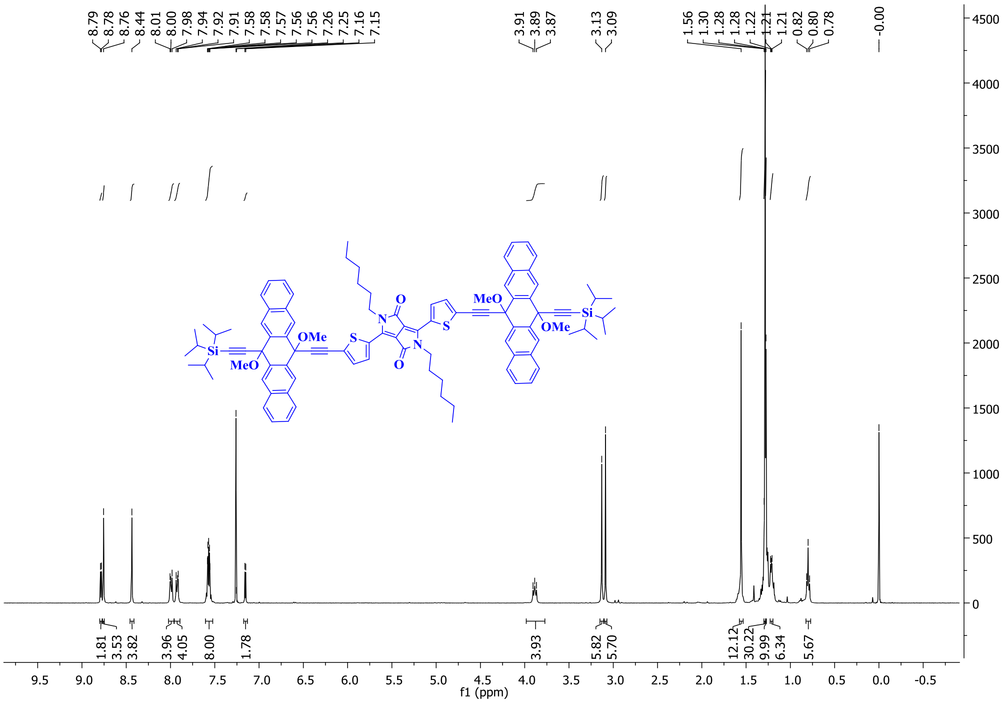

Supplementary Figure 26. ^{1}H NMR ($CDCl_3$, 400 MHz) of (OMe)2P-TDPP(OMe)

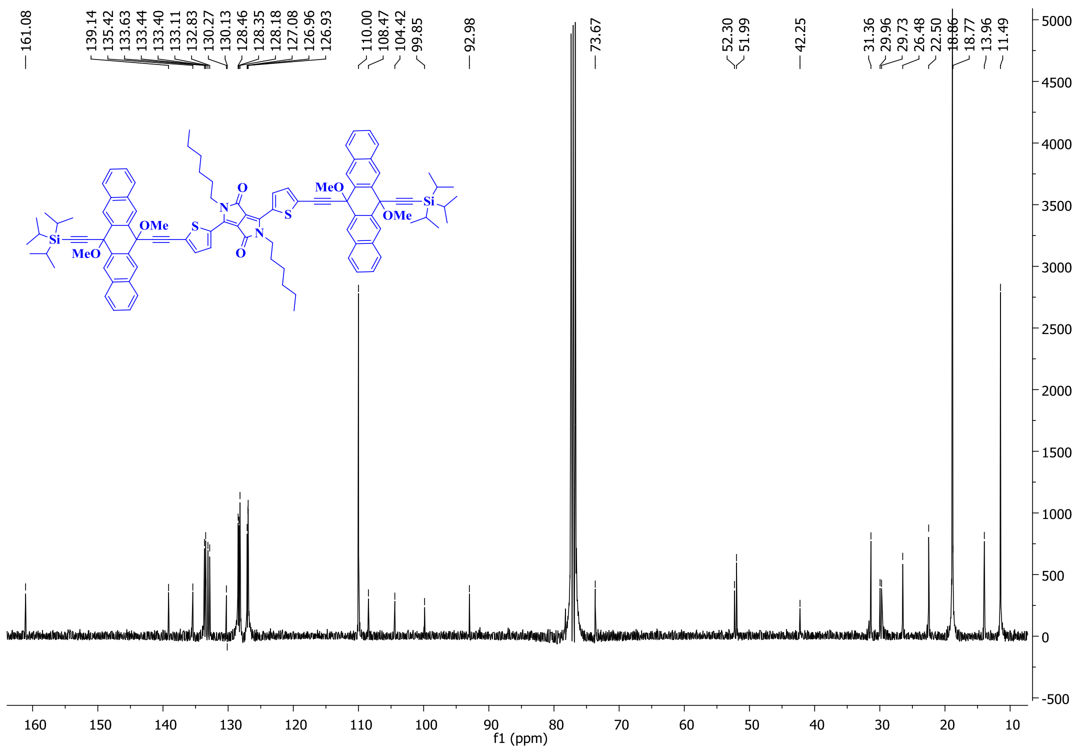

Supplementary Figure 27. ^{13}C NMR (125 MHz, $CDCl_3$) (OMe)2P-TDPP(OMe)

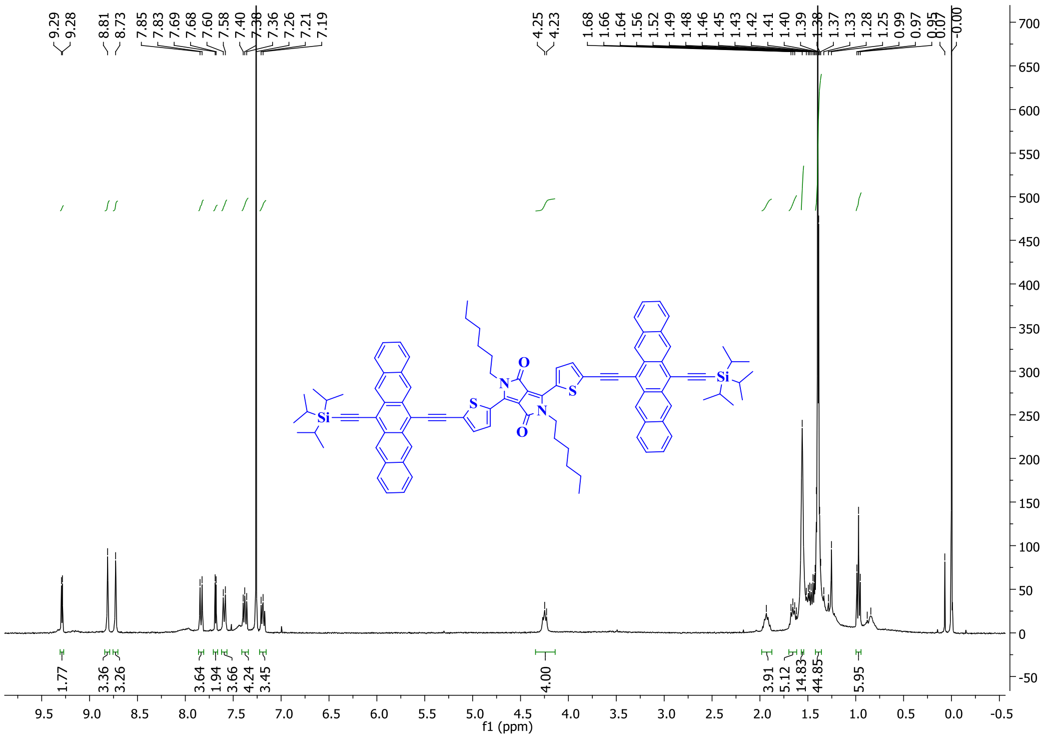

Supplementary Figure 28. ^{1}H NMR ($CDCl_3$, 400 MHz) of 2P-TDPP

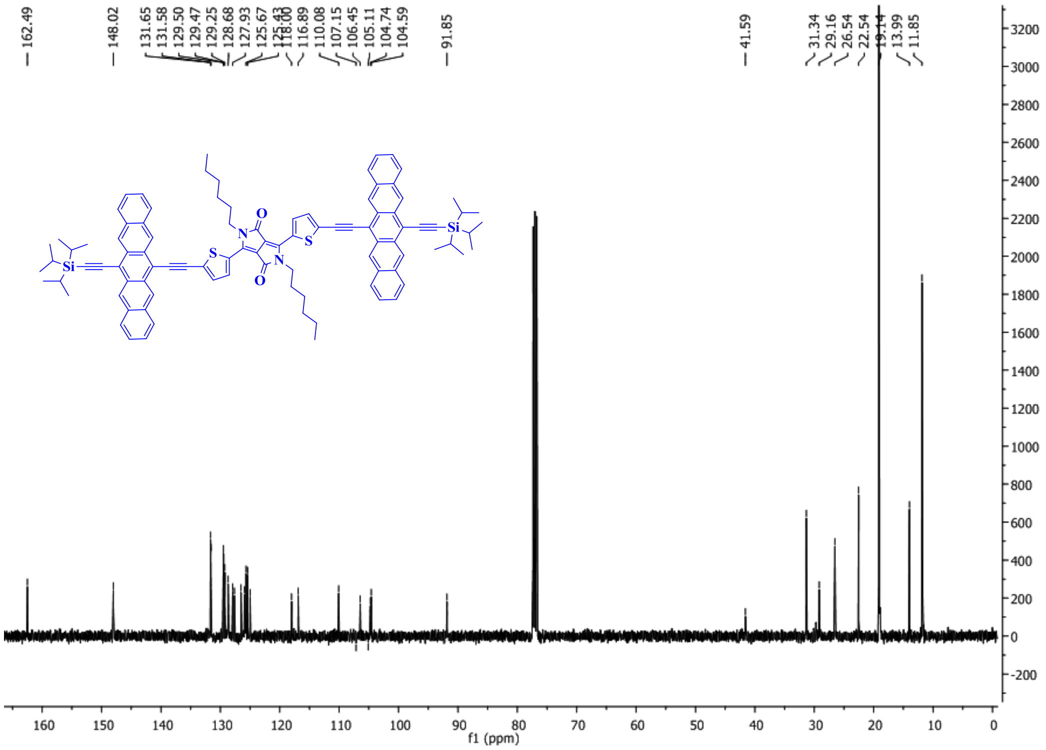

Supplementary Figure 29. ^{13}C NMR (125 MHz, $CDCl_3$) of 2P-TDPP

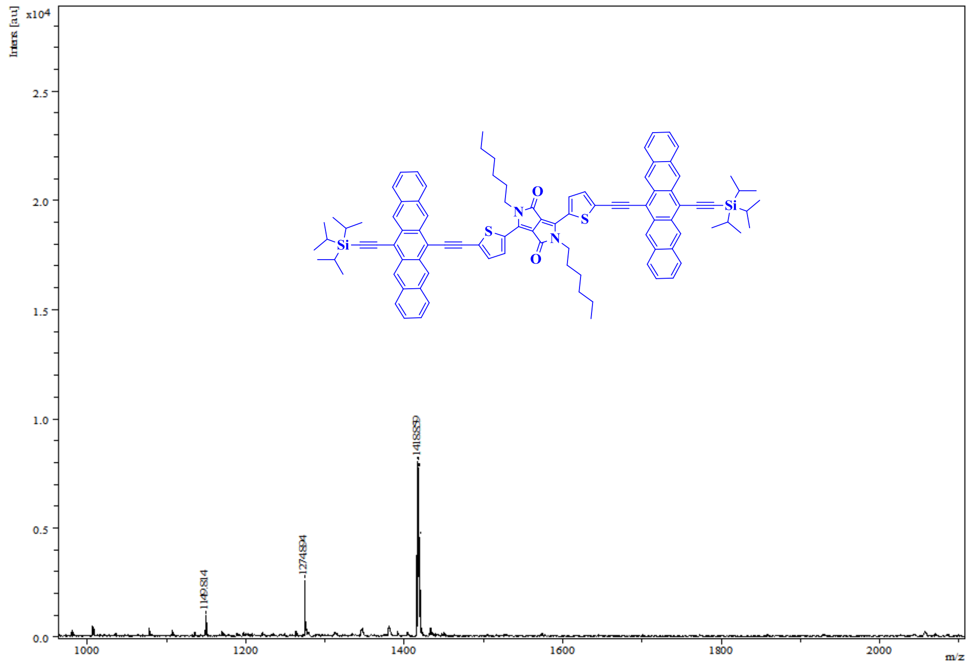

Supplementary Figure 30. MALDI-MS of 2P-TDPP

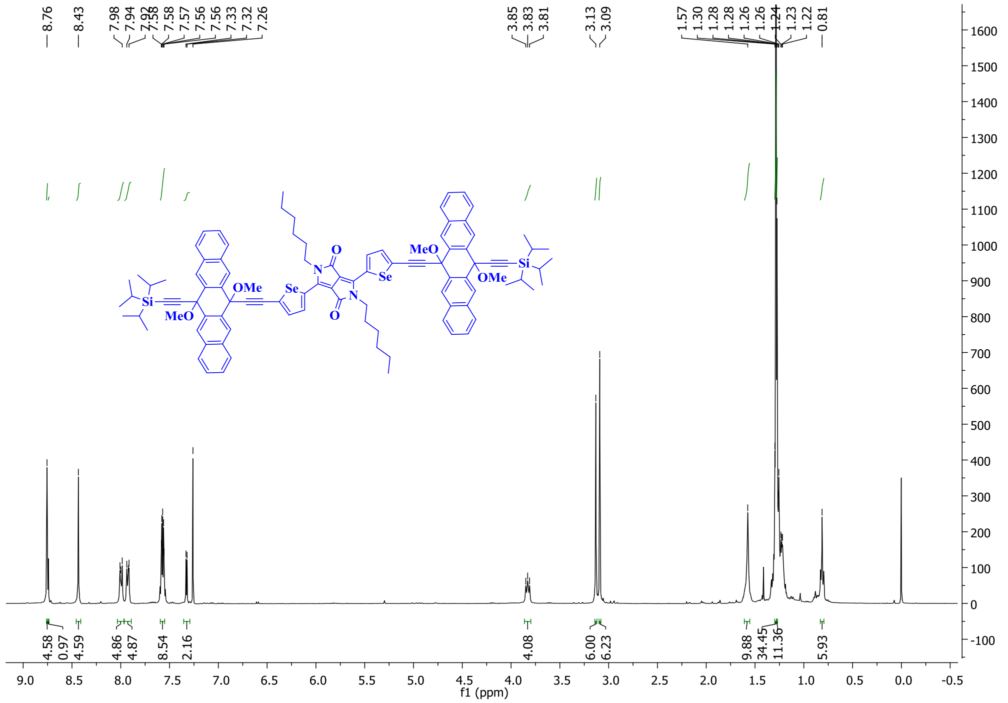

Supplementary Figure 31. ^{1}H NMR ($CDCl_3$, 400 MHz) of (OMe)2P-SeDPP(OMe)

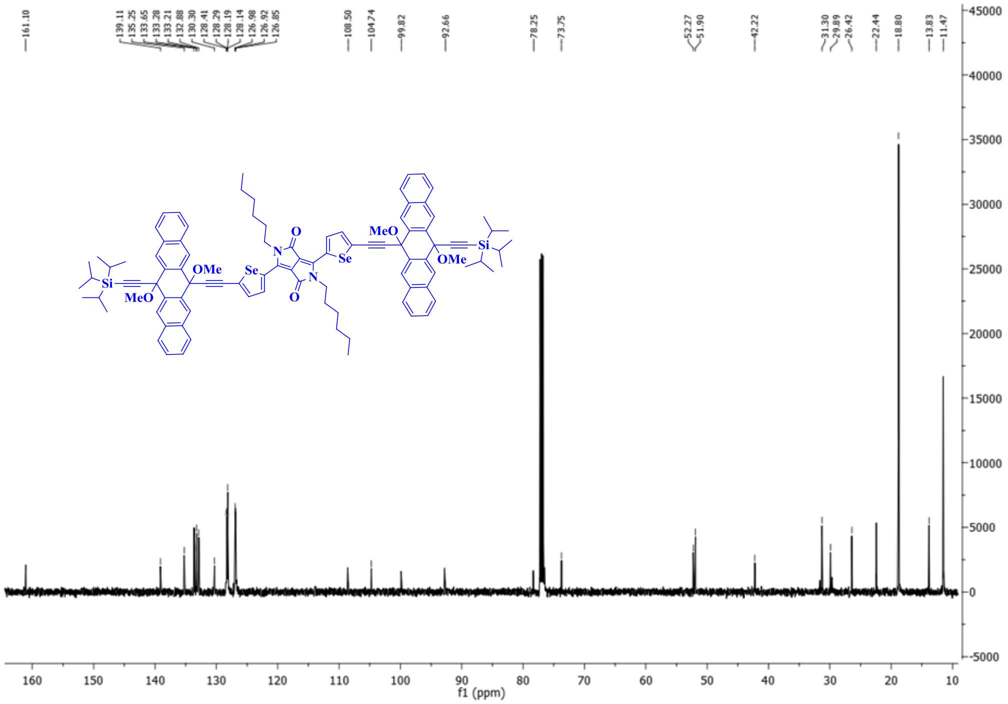

Supplementary Figure 32. ^{13}C NMR (125 MHz, $CDCl_3$) of (OMe)2P-SeDPP(OMe)

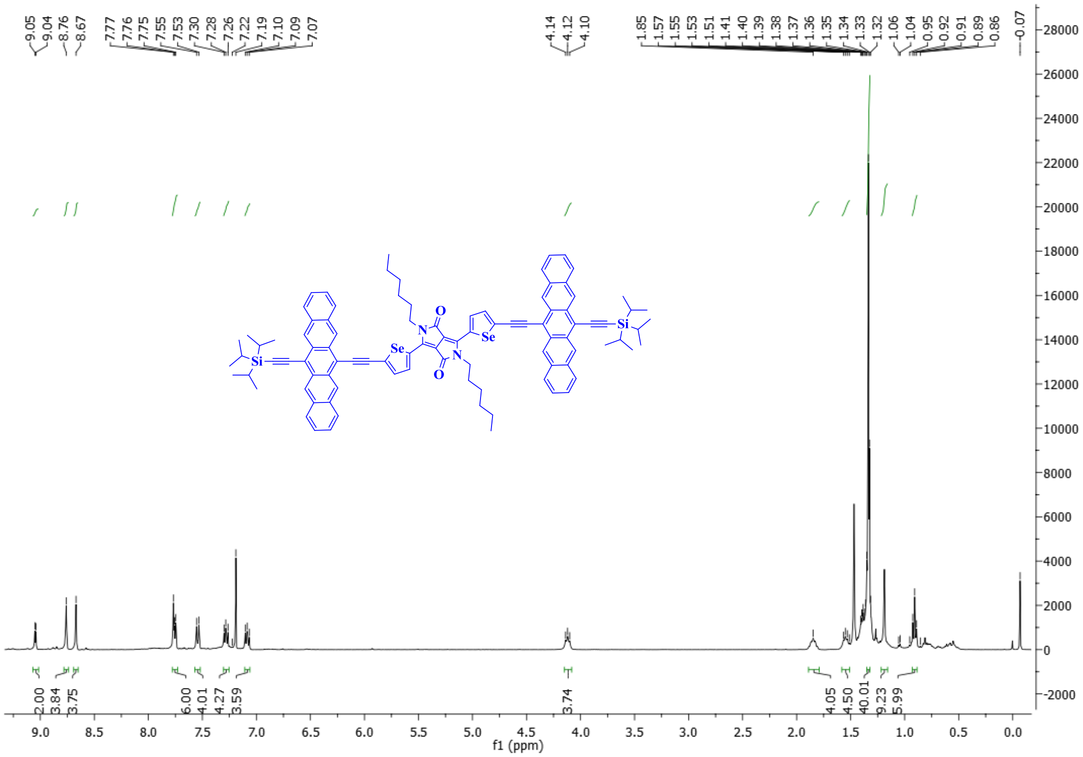

Supplementary Figure 33. ^{1}H NMR ($CDCl_3$, 400 MHz) of 2P-SeDPP

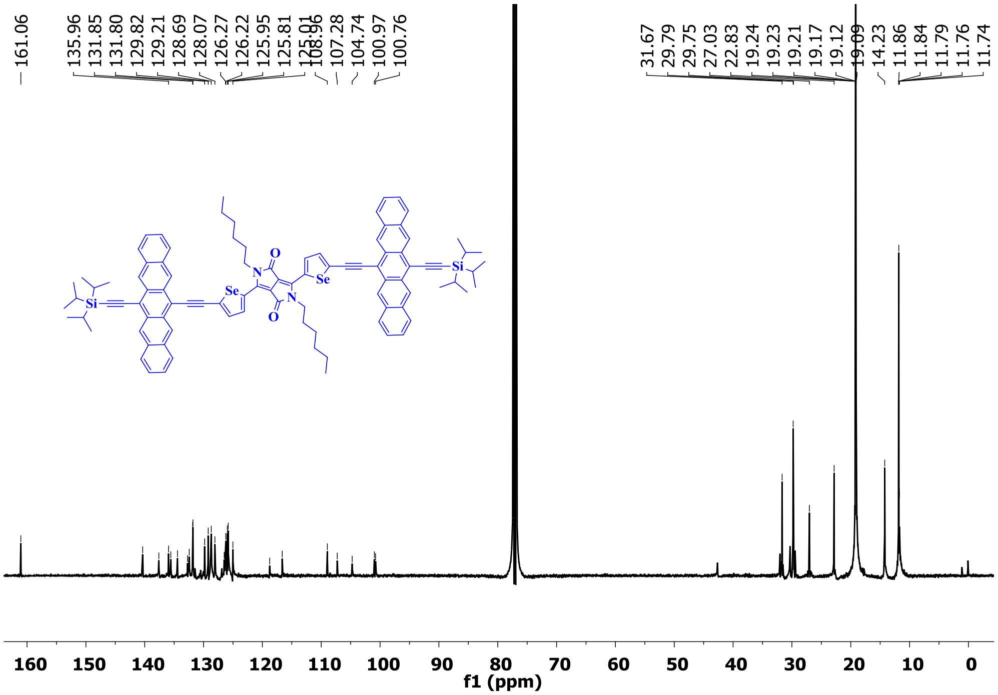

Supplementary Figure 34. ^{13}C NMR (125 MHz, $CDCl_3$) of 2P-SeDPP

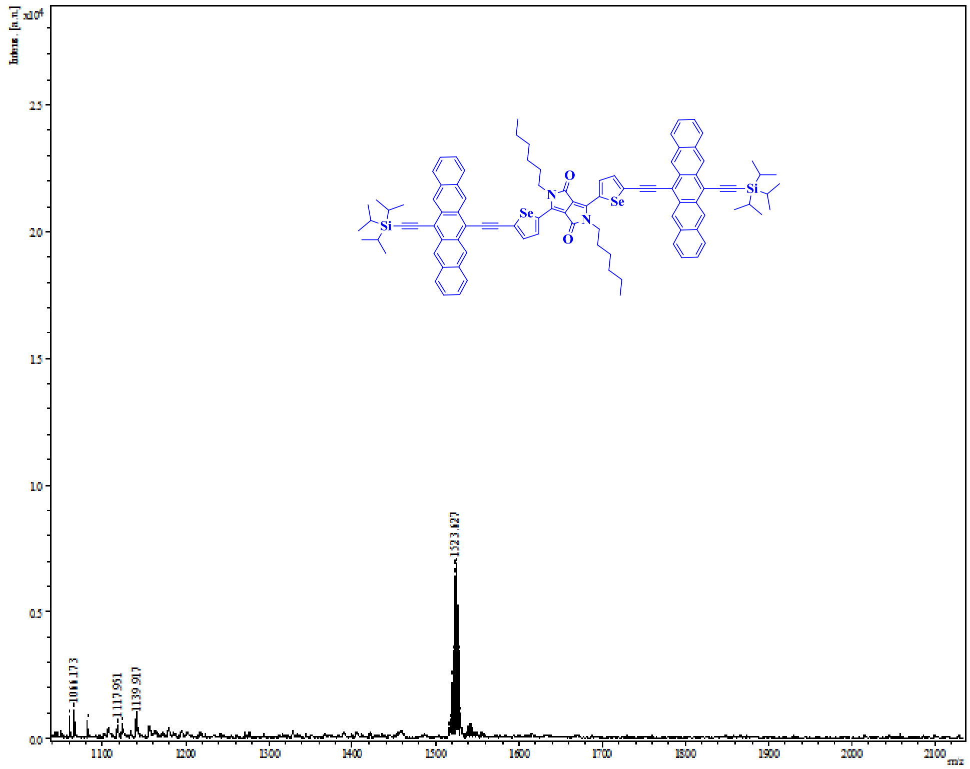

Supplementary Figure 35. MALDI-MS of 2P-SeDPP

Supplementary methods

1.1. Synthesis of (OMe)2P-PDPP (OMe) or 3,6-bis(4-((6,13-dimethoxy-13-((triisopropylsilyl)ethynyl)-6,13-dihydropentacen-6-yl)ethynyl)phenyl)-2,5-dihexylpyrrolo[3,4-c]pyrrole-1,4(2H,5H)-dione

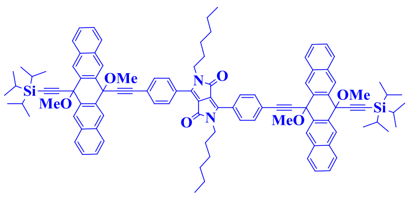

To a two-neck round bottomed flask [(13-ethynyl-6,13-dimethoxy-6,13-dihydropentacen-6-yl)ethynyl]triisopropylsilane (0.2g, 0.36mmol), 2,5-dihexyl-3,6-bis(4-iodophenyl)pyrrolo [3,4-c]pyrrole-1,4(*2H,5H*)-dione (0.13g, 0.18mmol), CuI (10mg) and THF/DIPA (1:1, 40mL) were added. The mixture was deoxygenated with argon for 20 min. $Pd(PPh_3)_4$ (15mg) was added under argon. The reaction mixture was maintained at 45°C for 24 h and then cooled down to room temperature. Water (100mL) was added and the resulting mixture was extracted with dichloromethane (3 x 50ml). The organic phase was dried over anhydrous Na_2SO_4 and filtered. After removing the solvent from the filtrate, the residue was purified by column chromatography on silica gel using ethyl acetate/hexane (2:8) as the eluent yielding an orange solid (0.25mg, 80%).

^{1}**H NMR** (400 MHz, $CDCl_3$), δ 8.79 (d, *J* = 4 Hz, 2H), 8.76 (s, 4H), 8.44 (s, 4H), 8.01-7.98 (m, 4H), 7.94-7.91 (m, 4H), 7.58-7.56 (m, 8H), 7.16-7.15 (d, 2H), 3.91-3.87 (m, 4H), 3.13 (s, 6H), 3.09 (s, 6H), 1.56 (br, s, 12H), 1.30-1.28 (m, 30H), 1.28 (br, s, 10H), 1.22-1.21 (m, 6H), 0.82-0.78 (t, *J* = 8 Hz, 6H) ppm.

^{13}**C NMR** (100MHz, $CDCl_3$) δ 162.49, 147.65, 133.94, 133.62, 133.18, 132.85, 131.96, 128.41, 128.32, 128.19, 127.68, 126.97, 126.81, 125.74, 106.08, 94.62, 92.34, 85.35, 73.61, 52.24, 52.01, 41.97, 31.14, 29.32, 22.41, 18.85, 18.78, 13.88, 11.48.

1.2. Synthesis of 2P-PDPP or 2,5-dihexyl-3,6-bis(4-((13-((triisopropylsilyl)ethynyl)pentacen-6-yl)ethynyl)phenyl)pyrrolo[3,4-c]pyrrole-1,4(2H,5H)-dione

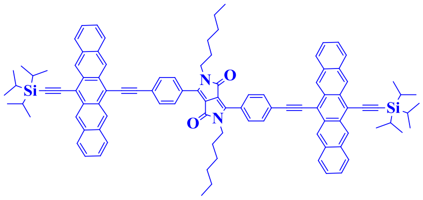

To a solution of 3,6-bis(4-((6,13-dimethoxy-13-((triisopropylsilyl)ethynyl)-6,13-dihydropentacen-6-yl)ethynyl)phenyl)-2,5-dihexylpyrrolo[3,4-c]pyrrole-1,4(*2H,5H*)-dione (0.2g, 0.13mmol) in dry THF (20mL) that had been deoxygenated by argon for 20 min $SnCl_2.H_2O$ (0.14g, 0.64mmol) was added followed by 10% aq H_2SO_4 (0.5mL). The reaction mixture was wrapped in aluminum foil to limit light exposure. This reaction mixture was further deoxygenated for 10 min. The reaction mixture was stirred at room temperature for 6 h. The resulting reaction mixture was filtered through pad of celite and the solvent was removed under reduced pressure. The crude product was purified by column chromatography on silica gel using ethyl acetate/hexane (1:9) as the eluent yielding a dark blue solid (0.13g, 70%).

^{1}**H NMR** (400 MHz, $CDCl_3$), δ 8.79 (d, *J* = 4 Hz, 2H), 8.88 (s, 4H), 8.76 (s, 4H), 7.99 (s, 8H), 7.84 (d, 8H), 7.38-7.34 (t, *J* = 8 Hz, 4H), 7.11-7.08 (t, *J* = 8 Hz, 4H), 4.02-3.98 (m, 4H), 1.63-1.62 (br, s, 6H), 1.49-1.47 (m, 30H), 1.30 (br, s, 6H), 0.92-0.89 (t, *J* = 8 Hz, 6H) ppm.

^{13}**C NMR** (100MHz, $CDCl_3$) δ 162.49, 148.02, 131.65, 131.58, 129.50, 129.50, 128.48, 127.93, 125.67, 125.43, 118.00, 116.89, 110.08, 107.15, 106.45, 105.11, 104.74, 104.59, 91.85, 41.59, 31.34, 29.16, 26.54, 22.54, 19.14, 13.99, 11.85.

MALDI-MS for $C_{120}H_{182}N_4O_4S_4$. *m/z* calculated: 1417.73; found: 1417.84

1.3. Synthesis of (OMe)2P-TDPP(OMe) or 3,6-bis(5-((6,13-dimethoxy-13-((triisopropylsilyl)ethynyl)-6,13-dihydropentacen-6-yl)ethynyl)thiophen-2-yl)-2,5-dihexylpyrrolo[3,4-c]pyrrole-1,4(2H,5H)-dione

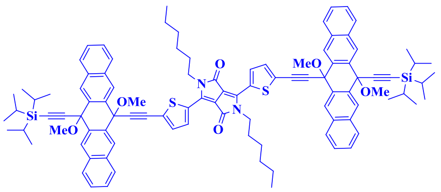

To a two-neck round bottomed flask [(13-ethynyl-6,13-dimethoxy-6,13-dihydropentacen-6-yl)ethynyl]triisopropylsilane (0.2g, 0.31mmol), 3,6-bis(5-bromothiophen-2-yl)-2,5-dihexylpyrrolo[3,4-c]pyrrole-1,4(*2H,5H*)-dione (0.12g, 0.19mmol), CuI (10mg) and THF/DIPA (1:1, 40mL) were added. The mixture was deoxygenated with argon for 20 min. $Pd(PPh_3)_4$ (15mg) was added under argon. The reaction mixture was maintained at 45 °C for 24 h and then cooled down to room temperature. Water (100mL) was added and the resulting mixture was extracted with dichloromethane (3 x 50ml). The organic phase was dried over anhydrous Na_2SO_4 and filtered. After removing the solvent from the filtrate, the residue was purified by column chromatography on silica gel using ethyl acetate/hexane (2:8) as the eluent yielding an orange solid (0.18mg, 75%).

^{1}H NMR (400 MHz, $CDCl_3$), δ 8.79 (d, *J* = 4 Hz, 2H), 8.76 (s, 4H), 8.44 (s, 4H), 8.01-7.99 (m, 4H), 7.94-7.91 (m, 4H), 7.58-7.56 (m, 8H), 7.16 (d, *J* = 4Hz, 2H), 3.91-3.87 (m, 4H), 3.13 (s, 6H), 3.09 (s, 6H), 1.56 (br, s, 12H), 1.30-1.28 (m, 30H), 1.28 (br, s, 10H), 1.22-1.21 (m, 6H), 0.82-0.78 (t, *J* = 8 Hz, 6H) ppm.

^{13}C NMR (100MHz, $CDCl_3$) δ 161.08, 139.14, 135.42, 133.63, 133.44, 133.40, 133.11, 132.83, 130.27, 130.13, 128.45, 128.35, 128.18, 127.08, 126.96, 126.93, 110.00, 108.47, 104.42, 99.85, 92.98, 73.67, 52.30, 51.99, 42.25, 31.36, 29.96, 29.73, 26.48, 22.50, 18.86, 18.77, 13.96, 11.49.

1.4. Synthesis of 2P-TDPP or 2,5-dihexyl-3,6-bis(5-((13-((triisopropylsilyl)ethynyl)pentacen-6-yl)ethynyl)thiophen-2-yl)pyrrolo[3,4-c]pyrrole-1,4(2H,5H)-dione

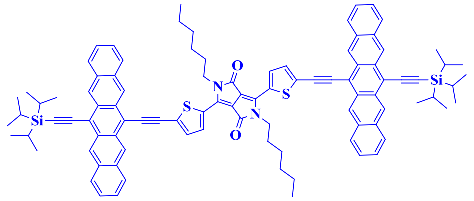

To a solution of 3,6-bis(5-((6,13-dimethoxy-13-((triisopropylsilyl)ethynyl)-6,13-dihydropentacen-6-yl)ethynyl)thiophen-2-yl)-2,5-dihexylpyrrolo[3,4-c]pyrrole-1,4(*2H,5H*)-dione (0.16g, 0.1mmol) in dry THF (20mL) that had been deoxygenated by argon for 20 min $SnCl_2.H_2O$ (0.11g, 0.51mmol) was added followed by 10% aq H_2SO_4 (0.5mL). The reaction mixture was wrapped in aluminum foil to limit light exposure. This reaction mixture was further deoxygenated for 10 min. The reaction mixture was stirred at room temperature for 6 h. The resulting reaction mixture was filtered through pad of celite and the solvent was removed under reduced pressure. The crude product was purified by column chromatography on silica gel using ethyl acetate/hexane (1:9) as the eluent yielding a dark green solid (0.13g, 70%).

^{1}**H NMR** (400 MHz, $CDCl_3$), δ 9.29 (d, *J* = 4 Hz, 2H), 8.81 (s, 4H), 8.73 (s, 4H), 7.85 (d, *J* = 8 Hz 4H), 7.69 (d, *J* = 4 Hz 2H), 7.60 (d, *J* = 8 Hz, 4H), 7.40 (t, *J* = 8 Hz, 4H), 7.21 (t, *J* = 8 Hz, 4H), 4.25-4.23 (m, 4H), 1.93 (br, s, 4H), 1.62-1.52 (m, 12H), 1.40 (br, s, 41H), 0.99 (t, *J* = 8 Hz, 6H) ppm.

^{13}**C NMR** (100 MHz, $CDCl_3$), δ 162.49, 148.02, 131.58, 129.50, 129.47, 129.25, 128.68, 127.93, 125.67, 125.43, 118.00, 116.89, 110.08, 107.15, 106.45, 105.11, 104.74, 104.59, 91.85, 41.59, 31.34, 29.16, 26.54, 22.54, 19.14, 13.99, 11.85.

MALDI-MS recorded for $C_{96}H_{96}N_2O_2S_2Si_2$. *m/z* calculated: 1429.64; found: 1429.70.

1.5. Synthesis of (OMe)2P-SeDPP(OMe) or 3,6-bis(5-((6,13-dimethoxy-13-((triisopropylsilyl)ethynyl)-6,13-dihydropentacen-6-yl)ethynyl)selenophen-2-yl)-2,5-dihexylpyrrolo[3,4-c]pyrrole-1,4(2H,5H)-dione

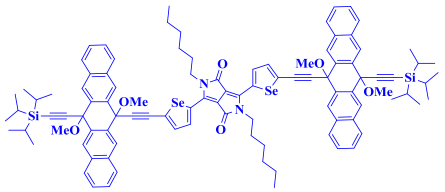

To a two-neck round bottomed flask [(13-ethynyl-6,13-dimethoxy-6,13-dihydropentacen-6-yl)ethynyl]triisopropylsilane (0.22g, 0.4mmol), 3,6-bis(5-bromoselenophen-2-yl)-2,5-dihexylpyrrolo[3,4-c]pyrrole-1,4(*2H,5H*)-dione (0.15g, 0.2mmol), CuI (10mg) and THF/DIPA (1:1, 40mL) were added. The mixture was deoxygenated with argon for 20 min. $Pd(PPh_3)_4$ (15mg) was added under argon. The reaction mixture was maintained at 45 °C for 24 h and then cooled down to room temperature. Water (100mL) was added and the resulting mixture was extracted with dichloromethane (3 x 50ml). The organic phase was dried over anhydrous Na_2SO_4 and filtered. After removing the solvent from the filtrate, the residue was purified by column chromatography on silica gel using ethyl acetate/hexane (2:8) as the eluent yielding an orange solid (0.25mg, 73%).

^{1}**H NMR** (400 MHz, $CDCl_3$), δ 8.76 (s, 5H), 8.74 (s, 1H), 8.43 (s, 4H), 8.01-7.98 (m, 4H), 7.94-7.92 (m, 4H), 7.58-7.56 (m, 8H), 7.33 (d, *J* = 8 Hz, 2H), 3.85-3.81 (m, 4H), 3.13 (s, 6H), 3.09 (s, 6H), 1.57 (br, s, 10H), 1.30-1.28 (m, 42H), 1.26 (br, s, 6H), 0.83 (t, *J* = 8 Hz, 6H) ppm.

^{13}**C NMR** (100 MHz, $CDCl_3$), δ 161.10, 139.11, 135.25, 133.65, 133.28, 133.21, 132.88, 130.30, 128.41, 128.29, 126.98, 126.92, 126.86, 108.50, 104.74, 99.82, 92.60, 73.75, 52.27, 51.90, 42.22, 31.30, 29.89, 26.42, 18.80, 13.83, 11.47.

1.6. Synthesis of 2P-SeDPP or 2,5-dihexyl-3,6-bis(5-((13-((triisopropylsilyl)ethynyl)pentacen-6-yl)ethynyl)selenophen-2-yl)pyrrolo[3,4-c]pyrrole-1,4(2H,5H)-dione

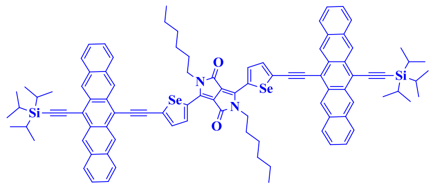

To a solution of 3,6-bis(5-((6,13-dimethoxy-13-((triisopropylsilyl)ethynyl)-6,13-dihydropentacen-6-yl)ethynyl)selenophen-2-yl)-2,5-dihexylpyrrolo[3,4-c]pyrrole-1,4(*2H,5H*)-dione (0.16g, 0.1mmol) in dry THF (20mL) that had been deoxygenated by argon for 20 min $SnCl_2.H_2O$ (0.1g, 0.48mmol) was added followed by 10% aq H_2SO_4 (0.5mL). The reaction mixture was wrapped in aluminum foil to limit light exposure. This reaction mixture was further deoxygenated for 10 min. The reaction mixture was stirred at room temperature for 6 h. The resulting reaction mixture was filtered through pad of celite and the solvent was removed under reduced pressure. The crude product was purified by column chromatography on silica gel using ethyl acetate/hexane (1:9) as the eluent yielding a dark green solid (0.10g, 68%).

^{1}**H NMR** (400 MHz, $CDCl_3$), δ 9.05 (d, *J* = 4 Hz, 2H), 8.76 (s, 4H), 8.87 (s, 4H), 7.89-7.87 (m, 8H), 7.77-7.75 (m, 6H), 7.55 (d, *J* = 8 Hz, 4H), 7.28 (t, *J* = 8 Hz, 4H), 7.09 (t, *J* = 8 Hz, 4H), 4.14-4.10 (m, 4H), 1.85 (br, s, 4H), 1.57-1.51 (m, 4H), 134-132 (m, 42H), 1.19 (br, s, 10H), 0.91 (t, *J* = 4Hz, 6H) ppm.

^{13}**C NMR** (100MHz, $CDCl_3$) δ 161.06, 135.96, 131.85, 131.80, 129.82, 129.21, 128.69, 128.07, 126.77, 126.22, 125.95, 125.81, 125.01, 108.96, 107.28, 104.79, 100.97, 100.76, 31.67, 29.79, 29.75, 27.03, 22.83, 19.24, 19.21, 19.17, 19.09, 14.23, 11.86.

MALDI-MS recorded for $C_{96}H_{96}N_2O_2Se_2Si_2$: *m/z* calculated: 1523.53; found: 1523.62

Supplementary References:

1. Dhar, J. *et al.* Photophysical, electrochemical and solid state properties of diketopyrrolopyrrole based molecular materials: importance of the donor group. *J. Mater. Chem. C* **2,** 3457–3466 (2014).

2. Lehnherr, D., Murray, A. H., McDonald, R. & Tykwinski, R. R. A Modular Synthetic Approach to Conjugated Pentacene Di-, Tri-, and Tetramers. *Angew. Chemie Int. Ed.* **49,** 6190–6194 (2010).

3. Lukman, S. *et al.* Tuning the role of charge-transfer states in intramolecular singlet exciton fission through side-group engineering. *Nat. Commun.* **7,** 13622 (2016).

4. Sanders, S. N. *et al.* Quantitative Intramolecular Singlet Fission in Bipentacenes. *J. Am. Chem. Soc.* **137,** 8965–8972 (2015).

5. Zirzlmeier, J. *et al.* Singlet fission in pentacene dimers. *Proc. Natl. Acad. Sci. U. S. A.* **112,** 5325–30 (2015).
